# A role for leucine-rich, glioma inactivated 1 in regulating pain sensitivity

**DOI:** 10.1093/brain/awae302

**Published:** 2024-09-20

**Authors:** Adham Farah, Ryan Patel, Piotr Poplawski, Benjamin J Wastie, Mandy Tseng, Allison M Barry, Omar Daifallah, Akash Dubb, Ivan Paul, Hoi lao Cheng, Faisal Feroz, Yuhe Su, Marva Chan, Hanns Ulrich Zeilhofer, Theodore J Price, David L Bennett, Kirsty Bannister, John M Dawes

**Affiliations:** Nuffield Department of Clinical Neurosciences, University of Oxford, Oxford OX3 9DU, UK; Wolfson Sensory, Pain & Regeneration Centre, Guy’s Campus, Kings College London, London SE1 1UL, UK; Nuffield Department of Clinical Neurosciences, University of Oxford, Oxford OX3 9DU, UK; Nuffield Department of Clinical Neurosciences, University of Oxford, Oxford OX3 9DU, UK; Nuffield Department of Clinical Neurosciences, University of Oxford, Oxford OX3 9DU, UK; Center for Advanced Pain Studies, School of Behavioral and Brain Sciences, Department of Neuroscience, University of Texas at Dallas, Richardson, TX 75080, USA; Nuffield Department of Clinical Neurosciences, University of Oxford, Oxford OX3 9DU, UK; Nuffield Department of Clinical Neurosciences, University of Oxford, Oxford OX3 9DU, UK; Nuffield Department of Clinical Neurosciences, University of Oxford, Oxford OX3 9DU, UK; Nuffield Department of Clinical Neurosciences, University of Oxford, Oxford OX3 9DU, UK; Nuffield Department of Clinical Neurosciences, University of Oxford, Oxford OX3 9DU, UK; Nuffield Department of Clinical Neurosciences, University of Oxford, Oxford OX3 9DU, UK; Nuffield Department of Clinical Neurosciences, University of Oxford, Oxford OX3 9DU, UK; Institute of Pharmacology and Toxicology, University of Zurich, 8057 Zurich, Switzerland; Institute of Pharmaceutical Sciences, Swiss Federal Institute of Technology (ETH) Zurich, 8093 Zurich, Switzerland; Center for Advanced Pain Studies, School of Behavioral and Brain Sciences, Department of Neuroscience, University of Texas at Dallas, Richardson, TX 75080, USA; Nuffield Department of Clinical Neurosciences, University of Oxford, Oxford OX3 9DU, UK; Wolfson Sensory, Pain & Regeneration Centre, Guy’s Campus, Kings College London, London SE1 1UL, UK; Nuffield Department of Clinical Neurosciences, University of Oxford, Oxford OX3 9DU, UK

**Keywords:** leucine-rich, glioma inactivated 1 (LGI1), mechanical pain hypersensitivity, neuropathic pain, hyperexcitability, wind-up

## Abstract

Neuronal hyperexcitability is a key driver of persistent pain states, including neuropathic pain. Leucine-rich, glioma inactivated 1 (LGI1) is a secreted protein known to regulate excitability within the nervous system and is the target of autoantibodies from neuropathic pain patients. Therapies that block or reduce antibody levels are effective at relieving pain in these patients, suggesting that LGI1 has an important role in clinical pain.

Here we have investigated the role of LGI1 in regulating neuronal excitability and pain-related sensitivity by studying the consequences of genetic ablation in specific neuron populations using transgenic mouse models.

LGI1 has been well studied at the level of the brain, but its actions in the spinal cord and peripheral nervous system are poorly understood. We show that *LGI1* is highly expressed in dorsal root ganglion (DRG) and spinal cord dorsal horn neurons in both mouse and human. Using transgenic mouse models, we genetically ablated *LGI1*, either specifically in nociceptors (*LGI1*^fl/*Nav1.8*+^) or in both DRG and spinal neurons (*LGI1*^fl/*Hoxb8*+^). On acute pain assays, we found that loss of *LGI1* resulted in mild thermal and mechanical pain-related hypersensitivity when compared with littermate controls. In *LGI1*^fl/Hoxb8+^ mice, we found loss of K_v_1 currents and hyperexcitability of DRG neurons. *LGI1*^fl/*Hoxb8*+^ mice displayed a significant increase in nocifensive behaviours in the second phase of the formalin test (not observed in *LGI1*^fl/*Nav1.8*+^ mice), and extracellular recordings in *LGI1*^fl/*Hoxb8*+^ mice revealed hyperexcitability in spinal dorsal horn neurons, including enhanced wind-up. Using the spared nerve injury model, we found that LGI1 expression was dysregulated in the spinal cord. *LGI1*^fl/*Nav1.8*+^ mice showed no differences in nerve injury-induced mechanical hypersensitivity, brush-evoked allodynia or spontaneous pain behaviour compared with controls. However, *LGI1*^fl/*Hoxb8*+^ mice showed a significant exacerbation of mechanical hypersensitivity and allodynia.

Our findings point to effects of LGI1 at the level of both the DRG and the spinal cord, including an important impact of spinal LGI1 on pathological pain. Overall, we find a novel role for LGI1 with relevance to clinical pain.

## Teaser

Using conditional genetic ablation in mice, Farah *et al.* show that disruption of LGI1 leads to increased pain sensitivity. Mice lacking *LGI1* display neuronal hyperexcitability, including enhanced wind-up of spinal neurons, as well as exacerbation of mechanical hypersensitivity following nerve injury.

## Introduction

Chronic pain is a major clinical problem. Neuropathic pain, i.e. pain arising as a result of damage or disease of the somatosensory nervous system, represents a sizeable proportion of chronic pain cases, affecting ∼8%–9% of the general population.^1,2^ Although analgesics are available, neuropathic pain is particularly resistant, and current therapies lack efficacy and are associated with significant side effects.^3^ This highlights the need to gain a better understanding of the pathological mechanisms that regulate pain sensitivity in order that better therapies can be developed in the future. We know that neuronal hyperexcitability forms the basis of many persistent pain conditions, including neuropathic pain. For instance, both preclinical and clinical studies show increased excitability of primary sensory neurons [whose cell bodies reside within the dorsal root ganglia (DRG)] following nerve injury,^4-6^ and blocking their activity can alleviate neuropathic pain in patients.^7^ The hyperexcitability of DRG neurons drives central alterations leading to an enhanced response of dorsal horn neurons to sensory stimuli termed central sensitization.^8^ These activity-dependent changes are accompanied by a loss of inhibitory tone in the spinal cord, contributing further to spinal hyperexcitability,^9^ and reducing the activity of certain dorsal horn neuron populations reduces neuropathic pain.^10^ This hyperexcitability is mediated by alterations in ion channels^11^; however, the molecular mechanisms that govern these changes, particularly in the clinical setting, remain to be understood fully.

Leucine-rich, glioma inactivated 1 (LGI1) is a neuronally secreted molecule important for protein–protein interactions in the nervous system. It is known to interact directly with catalytically inactive members of the disintegrin and metalloprotease protein (ADAM) family, specifically ADAM11, ADAM22 and ADAM23,^12^ and to form complexes with ion channels, such as voltage-gated potassium channel (K_v_)1 subunits and α-amino-3-hydroxy-5-methyl-4-isoxazolepropionic acid (AMPA) receptors, where it can regulate their cell surface expression.^13-16^ Disruption of LGI1 is linked to the development of hyperexcitability within the human nervous system, where loss-of-function mutations are found in patients with a rare form of inherited epilepsy,^17^ and autoantibodies (-Ab) targeting LGI1 are associated with autoimmune encephalitis, seizure development and pain.^18,19^ In line with this, preclinical studies have shown that disruption of LGI1, either through genetic ablation or patient-Abs, leads to abnormal synaptic transmission and hyperexcitability attributed to the dysregulation of presynaptic K_v_1 channels and postsynaptic AMPA receptors.^15,16,20^ In addition to synaptic function, LGI1 also regulates the intrinsic excitability of neurons, whereby LGI1-Ab treatment or genetic ablation results in a reduced firing threshold as a result of decreased K_v_1 current.^14,21^ Furthermore, application of LGI1 can reduce neuron excitability in a K_v_1-dependent manner.^14^ Interestingly, neuropathic pain is an increasingly recognized feature of LGI1 autoimmunity. It can be the sole presenting symptom and is particularly responsive to therapies that block or reduce the levels of antibodies,^19,22^ suggesting a causal link between LGI1 disruption and neuropathic pain in patients. Both K_v_1 channels and AMPA receptors play a role in pain signalling, and their dysregulation contributes to neuropathic pain in preclinical models.^23,24^ Given these observations and the pedigree of LGI1 in terms of governing neuronal excitability, we aimed to assess whether LGI1 also has a role in regulating pain sensitivity, using conditional genetic ablation in mice.

## Materials and methods

### Mouse lines

*LGI1* double floxed (*LGI1*^fl/fl^) mice^25^ were bred with either the *Nav1.8* Cre line^26^ or the *Hoxb8* Cre line^27^ to generate two conditional knockout lines, *LGI1*^fl/*Nav1.8*+/−^ and *LGI1*^fl/*Hoxb8*+/−^, respectively. In all mice, *LGI1* was double floxed with heterozygous Cre recombinase expression. Mice were maintained on a C57BL/6J strain background, with both male and female animals used between the ages of 6 and 16 weeks. Genotyping details can be found in the Supplementary material.

### Behavioural studies

Mice were acclimated to testing equipment and baseline values obtained by averaging data from three or four sessions. Testing was performed at a consistent time of day in the same designated behavioural suite. The experimenter was blind to genotype and handled the mice in a random order. Mice were assigned to group based on their genotype. In total, 29 *LGI1*^fl/*Nav1.8*−^, 37 *LGI1*^fl/*Nav1.8*+^, 47 *LGI1*^fl/*Hoxb8*−^ and 51 *LGI1*^fl/*Hoxb8*+^ mice were used in behavioural studies (82 males and 82 females).

### Histology

For immunohistochemistry and *in situ* hybridization (ISH) studies, mice were overdosed with pentobarbitone and transcardially perfused, initially with sterile saline, then with 20 ml of 4% paraformaldehyde (0.1 M phosphate buffer). Once dissected, the DRG were post-fixed in 4% paraformaldehyde for 1.5 h at room temperature and spinal cord overnight at 4°C. All tissue was dehydrated for cyroprotection in 30% sucrose (0.1 M phosphate buffer) at 4°C for ≥24 h. Tissue was then embedded in optimal cutting temperature (O.C.T.) medium (Tissue-Tek) and stored at −80°C. Tissue was sectioned onto SuperFrost Plus slides (VWR) using a cryostat. DRG sections were cut at 10 μm thickness and spinal cord at 20 μm thickness. The slides were then stored at −80°C.

### *In vitro* whole-cell patch-clamp electrophysiology

Whole-cell patch-clamp recordings were performed on cultured mouse DRG neurons at room temperature (22°C) using an Axopatch 200B amplifier and Digidata 1550 acquisition system (Molecular Devices). Both current- and voltage-clamp recordings were performed.

### Extracellular dorsal horn neuron electrophysiology

*In vivo* electrophysiology was performed as previously described.^28^ Extracellular recordings were made from deep dorsal horn wide dynamic range (WDR) laminae V/VI neurons with receptive fields on the glabrous skin of the toes using 127 μm, 2 MΩ parylene-coated tungsten electrodes (A-M Systems). Searching involved light tapping of the hindpaw whilst manually moving the electrode. All recordings were made at depths delineating the deep dorsal horn laminae^29^ and were classified as WDR on the basis of neuronal sensitivity to dynamic brushing (i.e. gentle stroking with a #2 squirrel-hair artist’s brush) and noxious punctate mechanical (15 g) and heat (48°C) stimulation of the receptive field. One to three neurons were characterized per mouse; in total, 16 neurons were characterized from 13 *LGI1*^fl/Hoxb8−^ and 16 neurons from 13 *LGI1*^fl/Hoxb8+^ mice.

### Statistical analysis

For all other experiments, animals were used as the experimental unit. Sample sizes for each experiment can be found in the figure legends. Sample sizes were based on previous experience and power calculations conducted using the Gpower software with a *P*-value of 0.05 and with a power of >0.8. Note that multiple cohorts were used for behaviour studies and combined for final analysis. Significance for all experiments was placed at *P* < 0.05. Statistical tests were carried out with Sigmaplot and Graphpad Prism.

Additional details on all methods used can be found in the Supplementary material.

## Results

### *LGI1* is highly expressed in DRG and spinal cord

**Figure 1:**
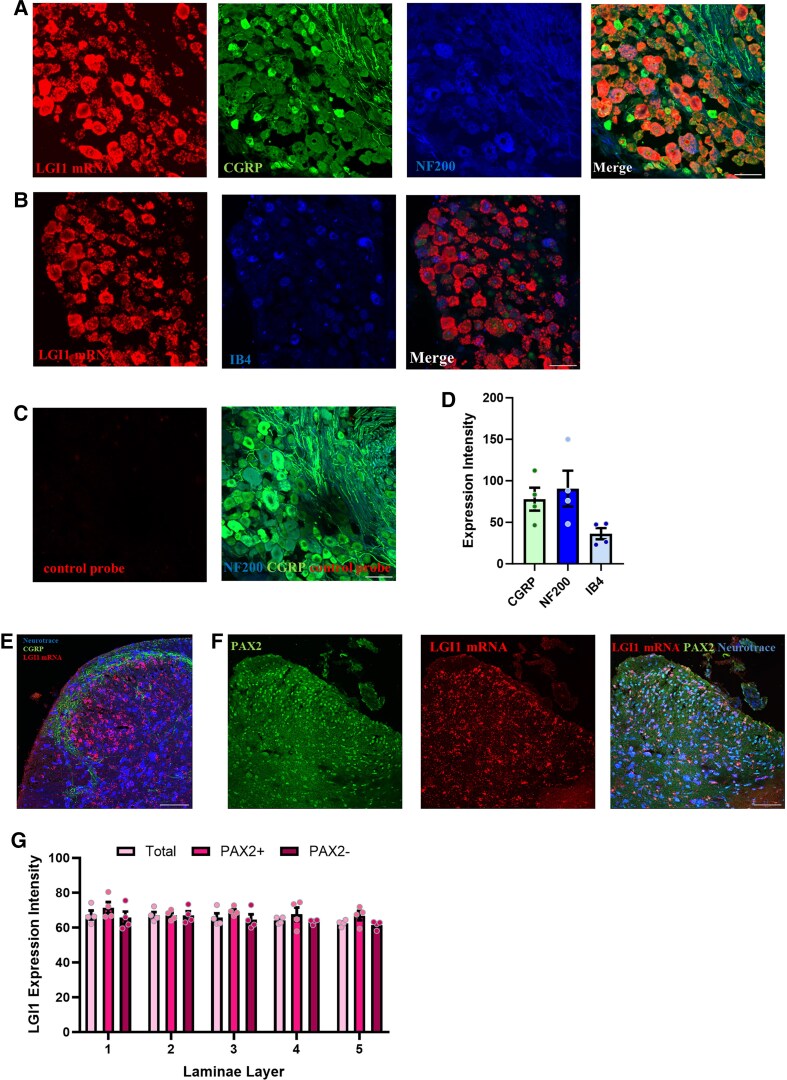
***LGI1***  **expression in dorsal root ganglion (DRG) and spinal cord.** (**A** and **B**) Representative images of mouse L4 DRG showing *in situ* hybridization (ISH) for *LGI1* mRNA (red) in CGRP+ (green), NF200+ (blue) (**A**) and IB4+ neurons (**B**); scale bar = 50 µm. (**C**) No ISH signal is observed in DRG neurons when using a negative control probe. (**D**) Quantification of *LGI1* mRNA signal intensity in DRG neurons (*n* = 3 or 4 mice). (**E**) Representative images of mouse lumbar spinal cord showing ISH for *LGI1* mRNA (red), with Neurotrace (blue) to mark neurons and CGRP (green) to mark lamina IIo; scale bar = 100 µm. (**F**) Representative images of mouse lumbar spinal cord showing ISH for *LGI1* mRNA (red), with Neurotrace to mark all neurons (blue) and Pax-2 (green) to mark inhibitory neurons; scale bar = 100 µm. (**G**) Quantification of *LGI1* mRNA signal intensity in mouse lumbar spinal cord (*n* = 4 mice). All data are shown as the mean ± standard error of the mean (SEM).

Few studies have assessed the expression of LGI1 in regions of the nervous system outside of the brain. Given the link to neuropathic pain, we first wanted to analyse the expression of *LGI1* in both the DRG and spinal cord dorsal horn (SCDH), two key areas in pain signalling. Using RNA ISH, we found high levels of *LGI1* mRNA expressed within neurons of both the mouse lumbar DRG and SCDH (Fig. 1A–G). In combination with immunohistochemistry, markers were used to define broad populations of DRG neurons. Highest *LGI1* mRNA expression was seen in neurons that co-expressed either NF200 (a marker of Aβ and Aδ myelinated fibres) or CGRP (a marker of peptidergic nociceptors) (Fig. 1A and D). Expression was also seen in neurons that bind the lectin IB4 (marker of non-peptidergic nociceptors), albeit to a lower level (Fig. 1B and D). No mRNA signal was detected when using a negative control probe (Fig. 1C). In the SCDH, *LGI1* mRNA levels were broadly similar across superficial and deeper lamina (Fig. 1E–G). To gain insight into the types of neurons expressing *LGI1*, we assessed neurons for their expression of Pax-2 (a marker of inhibitory interneurons). We found similar levels of *LGI1* in both Pax-2+ and Pax-2− (putative excitatory) neurons in the SCDH (Fig. 1F and G).

Given that the interaction of LGI1 with K_v_1 channels and AMPA receptors occurs through ADAM proteins, we used ISH to also assess their expression. We found that *ADAM11*, *ADAM22* and *ADAM23* were expressed in mouse lumbar DRG, following a similar pattern to that of *LGI1*, with highest levels in NF200+ neurons (Supplementary Fig. 1A–D). *ADAM11* and *ADAM22* showed highest expression in nociceptor populations (Supplementary Fig. 1A, B and D). All three were well expressed in the mouse lumbar SCDH, with similar levels in both Pax-2+ and Pax-2− neurons (Supplementary Fig. 1E–H).

To gain an idea of how DRG and spinal cord levels of *LGI1*, *ADAM11*, *ADAM22* and *ADAM23* compared with the whole brain, we assessed relative expression using qPCR. For *LGI1*, similar levels were seen across the three tissues; however, the highest expressions for *ADAM11*, *ADAM22* and *ADAM23* were found in the DRG compared with spinal cord and brain (Supplementary Fig. 1I). Using previously published single-nucleus and spatial RNA-sequencing (RNA-Seq) datasets, we also explored the expression of *LGI1* and interaction partners in human tissue (Supplementary Fig. 2). In human DRG,^30^ the expression of *LGI1* is present across subpopulations, with higher levels in neurons enriched for pruritogen receptors, silent nociceptors and Aβ nociceptors (Supplementary Fig. 2D). Here, the co-expression with interaction partners is also seen. In human spinal cord,^31^  *LGI1* has overlapping expression with *ADAM22* and *ADAM23*, with a lack of expression in motor neurons (Supplementary Fig. 2A–C). At the neuronal subtype level, these genes are expressed in a mix of inhibitory and excitatory neurons (Supplementary Fig. 2A–C), similar to that seen in the mouse (Fig. 1E–G and Supplementary Fig. 1E–H). These data show that *LGI1* and its known binding partners, *ADAM11*, *ADAM22* and *ADAM23*, are highly expressed in pain-relevant regions of the nervous system (e.g. DRG and SCDH) in both mouse and human.

### *LGI1* ablation leads to pain-related hypersensitivity in mice

To investigate whether the expression of LGI1 in the DRG and SCDH had functional significance in relationship to regulating pain sensitivity, we generated conditional knockout mice using the *Hoxb8* Cre line (*LGI1*^fl/*Hoxb8*+^), where Cre recombinase expression is restricted anatomically to caudal DRG and spinal cord.^27^ We confirmed LGI1 removal from DRG and spinal cord at lumbar and thoracic levels using qPCR (Supplementary Fig. 3A and B). Residual expression remained in cervical DRG and spinal cord, with normal levels found in the brain (Supplementary Fig. 3A and B). Global ablation of LGI1 results in seizure development.^15,25^ Owing to the preservation of LGI1 in the brain, we observed no seizures in these mice.

There are four members of the LGI family (LGI1–LGI4).^32^ Using qPCR, we also assessed the expression levels of *LGI2–LGI4* and of *ADAM11*, ADAM*22* and *ADAM23* for any compensatory expression changes following removal of *LGI1*. Except for a small but significant decrease in *ADAM11* expression in cervical DRG and spinal cord of *LGI1*^fl/*Hoxb8*+^ mice, expression levels of these genes, on the whole, were not statistically significantly affected following ablation of *LGI1* (Supplementary Table 1).

**Figure 2:**
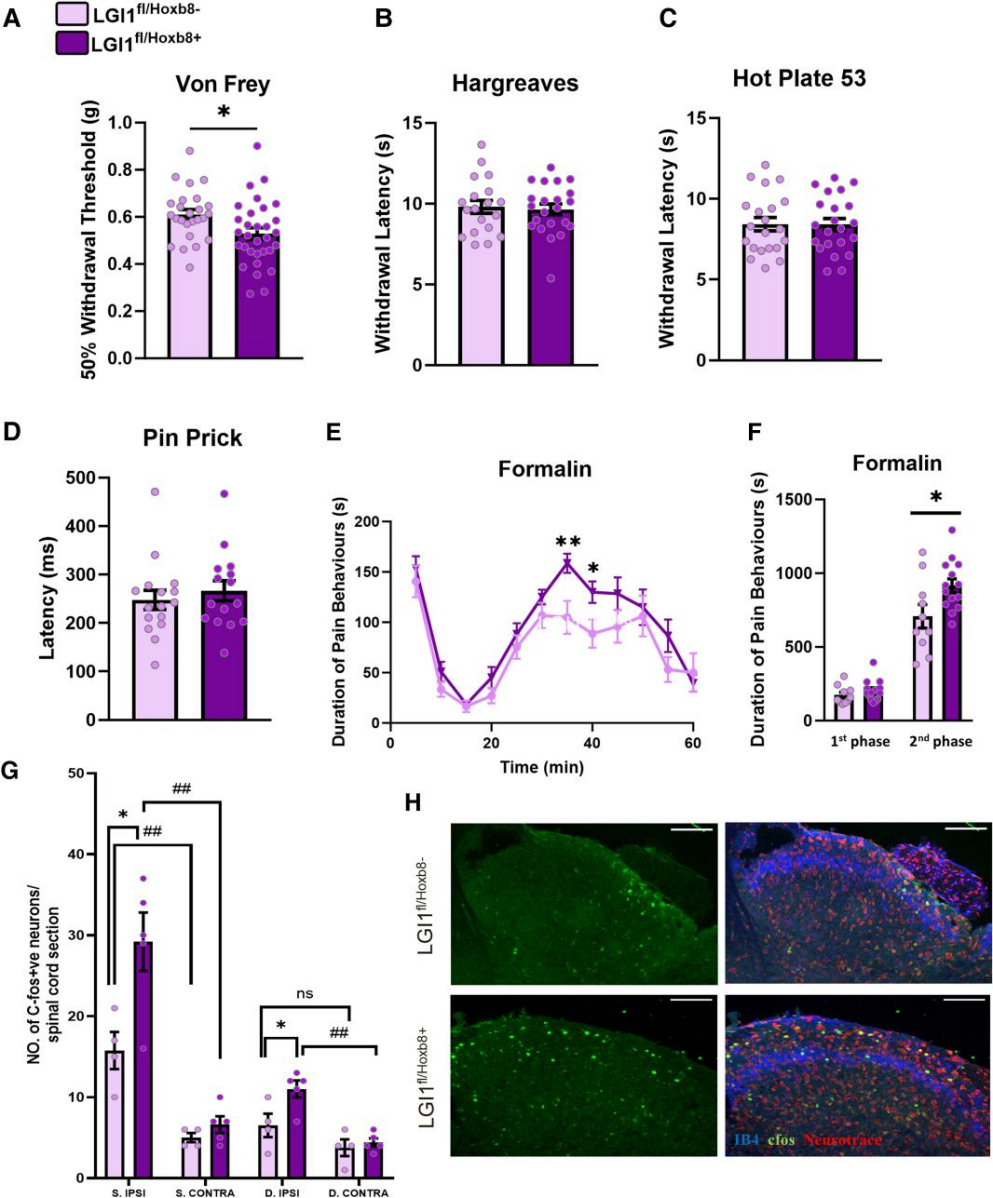
**Conditional ablation of *LGI1* increases pain-related behaviour in mice.** (**A**) Using Von Frey hair application, *LGI1*^fl/*Hoxb8*+^ mice (*n* = 31) displayed mechanical pain-related hypersensitivity when compared with littermate controls (*n* = 25). (**B** and **C**) *LGI1*^fl/*Hoxb8*+^ mice did not display thermal hypersensitivity as measured by the Hargreaves test (**B**) and/or a hot plate set at 53°C (**C**) (*LGI1*^fl/*Hoxb8*+^  *n* = 22; *LGI1*^fl/*Hoxb8*−^, *n* = 21). (**D**) Withdrawal latency to pin prick application was not altered in *LGI1*^fl/*Hoxb8*+^ mice (*n* = 15) versus controls (LGI1^fl/*Hoxb8*−^, *n* = 16). (**E**) *LGI1*^fl/*Hoxb8*+^ mice (*n* = 14) display increased nocifensive behaviour during the second phase of the formalin test when compared with littermate controls (*n* = 10). (**F**) Total duration of nocifensive behaviour during both the first phase (0–15 min) and the second phase (15–60 min) of the formalin test. (**G**) The number of c-Fos^+^ neurons is increased in the ipsilateral superficial dorsal horn (laminae I + II, S. IPSI) and deeper laminae (III + IV, D. IPSI) of the spinal cord in *LGI1*^fl/*Hoxb8*+^ mice 2 h after formalin injection (*n* = 5) compared with littermate controls (*n* = 4). An increase in ipsilateral c-Fos expression is seen for both genotypes when compared with contralateral (CONTRA) spinal cord. (**H**) Representative images of c-Fos expression (green) in the ipsilateral spinal cord dorsal horn of *LGI1*^fl/*Hoxb8*−^ and *LGI1*^fl/*Hoxb8*+^ mice following formalin injection. Neurons are marked with Neurotrace (red), with IB4 shown in blue; scale bar = 100 µm. Data are shown as the mean ± SEM; *P < 0.05 and **P < 0.01 versus *LGI1*^fl/*Hoxb8*−^; ^#^*P* < 0.05 and ^##^*P* < 0.01 versus contralateral. ns = not significant.

We therefore next assessed how specific removal of *LGI1* from DRG and spinal cord impacted on pain-related behaviour. Compared with littermate controls (*LGI1*^fl/*Hoxb8*−^), genetic removal of *LGI1* resulted in mechanical pain-related hypersensitivity as measured by Von Frey hair application, with a small but significant reduction in withdrawal thresholds (*LGI1*^fl/*Hoxb8*−^ 0.61 ± 0.02 g versus *LGI1*^fl/*Hoxb8*+^ 0.53 ± 0.04 g; Fig. 2A). No differences were observed between groups for thermal sensitivity by measuring response latencies to both a radiant heat source (Hargreaves method) and a hot plate set at 53°C (Fig. 2B and C) or for the withdrawal response to pin prick application (Fig. 2D). When assessing non-pain-related behaviour, there was no difference between genotypes in the detection of innocuous mechanical stimuli (Supplementary Fig. 4A and B).

We also studied proprioception and motor activity at the behavioural level, with no significant differences observed in the performance of *LGI1*^fl/*Hoxb8*+^ mice compared with controls (Supplementary Fig. 4C–F). For comparison, we also generated a nociceptor-specific knockout line (*LGI1*^fl/*Nav1.8*+^) using *Nav1.8* Cre mice^26^ (Supplementary Fig. 3C). When compared with littermate controls, *LGI1*^fl/*Nav1.8*+^ mice showed no differences in mechanical sensitivity (Supplementary Fig. 5A and D) or thermal sensitivity using the hot plate test (Supplementary Fig. 5C). A reduced latency to withdrawal was seen for *LGI1*^fl/*Nav1.8*+^ mice following radiant heat source stimulation (Supplementary Fig. 5B). These findings show that genetic removal of *LGI1* has a subtle, but significant impact on acute pain-related thresholds, albeit dependent on the conditional knockout line used.

We next wanted to assess the consequences of *LGI1* removal in the context of a more tonic pain state. To do this, we used formalin injection, which results in a biphasic overt pain behaviour, attributed to both peripheral (first phase) and central (second phase) mechanisms.^33,34^ We saw no differences between genotypes in the first phase of behaviour. However, *LGI1*^fl/*Hoxb8*+^ mice displayed significantly enhanced nocifensive behaviour in the second phase following formalin injection when compared with littermate controls (*LGI1*^fl/*Hoxb8*−^ 708.5 ± 79.9 s versus *LGI1*^fl/*Hoxb8*+^ 916.5 ± 45.7 s; Fig. 2E and F), whereas no differences in the response to formalin was observed when *LGI1* was ablated specifically in nociceptors (Supplementary Fig. 5E and F). Assessment of c-Fos expression revealed that the enhanced second phase of the formalin test following ablation of *LGI1* from spinal neurons was correlated with a significant increase in the activation of neurons in both superficial and deeper laminae of the lumbar SCDH when compared with controls (Fig. 2G and H).

It is now recognized that sex-specific mechanisms can contribute to pain.^35^ However, we saw no effect of sex on acute pain behaviour or the response to formalin, suggesting that the development of pain hypersensitivity following *LGI1* ablation is relevant to both sexes (Supplementary Tables 2 and 3). Of note, RNA-Seq data from human DRG showed similar levels of *LGI1* expression in both males and females (Supplementary Fig. 2E; log fold change = 0.13, false discovery rate = 0.38).

### The effects of *LGI1* ablation on DRG neuron excitability

**Figure 3:**
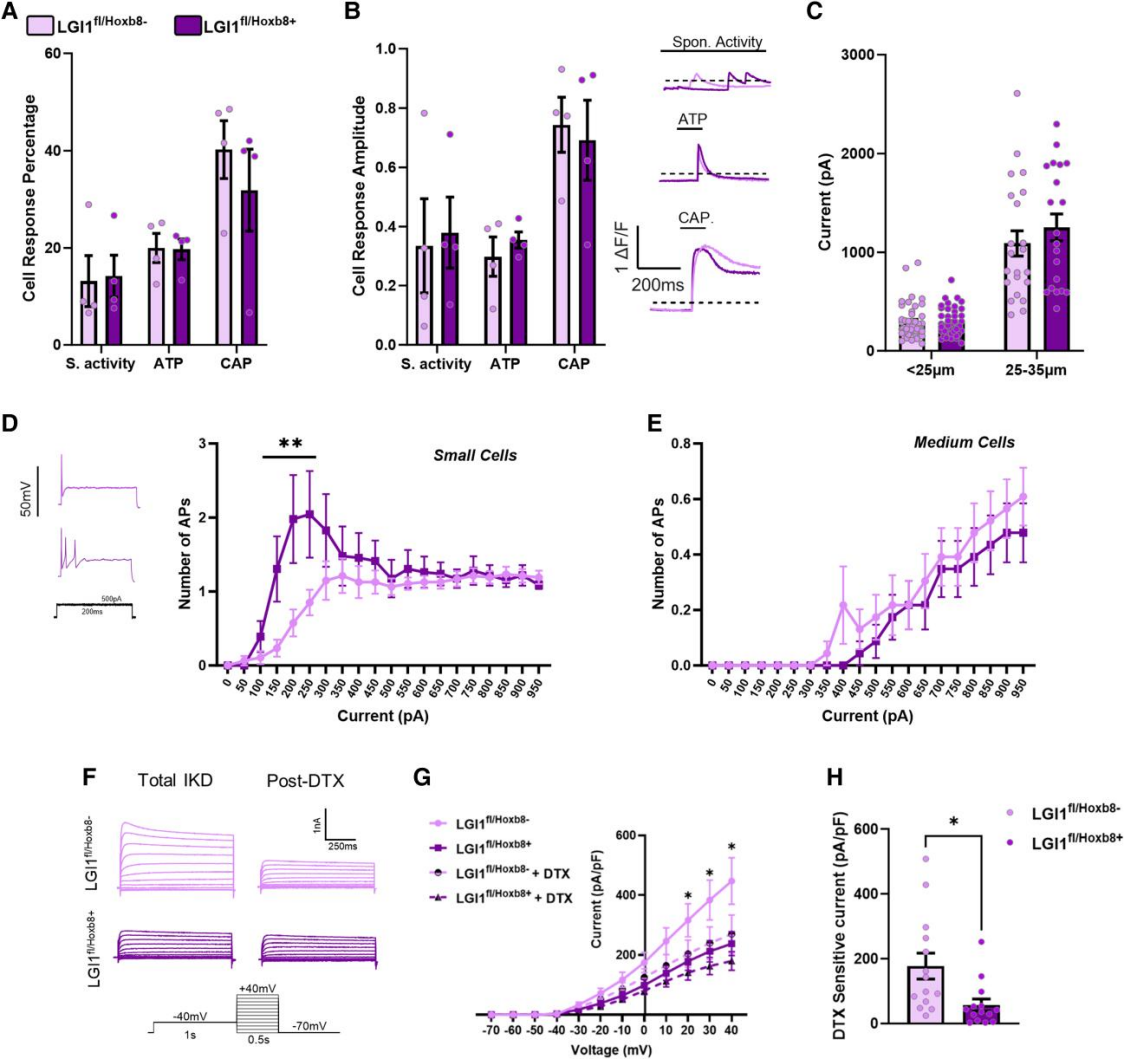
**Dorsal root ganglion (DRG) neuron excitability in *LGI1*^fl/*Hoxb8*+^ mice.** (**A** and **B**) Calcium imaging data for DRG neurons cultured from *LGI1*^fl/*Hoxb8*+^ and *LGI1*^fl/*Hoxb8*−^ mice (*n* = 4 mice, data taken from ∼500 cells per genotype) during the initial untreated period [spontaneous (S) activity] and in response to both ATP (10 μM) and capsaicin (CAP, 1 μM). No difference was seen between genotypes for both the percentage of DRG neurons responding (**A**) and the amplitude of their response (**B**, *left*). Neurons were identified by their response to 50 mM KCl. (**B**, *right*) Example traces of calcium transients. (**C**) Whole-cell patch-clamp electrophysiology revealed no difference in the rheobase of both small (<25 μm in diameter, *n* = 46 or 47 cells taken from four mice) or medium-sized (25–35 μm, *n* = 23 taken from four mice) DRG neurons between genotypes. (**D** and **E**) Action potential firing in response to a range of prolonged current injections in both small cells (**D**) and medium cells (**E**). (**D**) Small DRG neurons from *LGI1*^fl/Hoxb8+^ mice displayed significantly increased firing in comparison to controls. Representative traces are also shown. (**F**) Example traces of outward current in small DRG neurons (<25 µm in diameter) from both *LGI1*^fl/*Hoxb8*+^ and *LGI1*^fl/*Hoxb8*−^ mice evoked by depolarizing pulses. (**G**) *I*_KD_ (slowly-inactivating potassium current) was measured before and after 100 nM dendrotoxin (DTX). Current–voltage relationships for *I*_KD_ pre-DTX (total *I*_KD_) were significantly reduced in *LGI1*^fl/*Hoxb8*+^ neurons (*n* = 14) compared with controls (*n* = 14). (**H**) DTX-sensitive currents (total *I*_KD_ − *I*_KD_ + DTX) were significantly reduced in small-diameter neurons from *LGI1*^fl/*Hoxb8*+^ mice. All data are shown as the mean ± SEM. (**D**) **P < 0.01 versus *LGI1*^fl/*Hoxb8*−^ neurons. (**G**) *P < 0.05 versus *LGI1*^fl/*Hoxb8*+^.

Given the role of LGI1 in regulating excitability in relationship to epilepsy within the nervous system, we next investigated whether neuron activity in pain circuits was altered in our conditional *LGI1* knockout lines. Beginning at the level of the DRG, we used *in vitro* calcium imaging to measure the sensitivity of neurons from both *LGI1*^fl/*Hoxb8*+^ and control mice. In terms of the percentage of cells responding, no differences were seen between genotypes when cells were not stimulated (as a measure of spontaneous activity) or following application of the chemical algogens capsaicin and ATP, used to activate specific nociceptor populations (Fig. 3A). Nor were there any differences in the amplitude of response (Fig. 3B). Likewise, no differences in the percentage of cells responding or response amplitudes were measured in cultured DRG neurons from *LGI1*^fl/*Nav1.8*+^ mice versus controls (Supplementary Fig. 5G and H).

We next used *in vitro* whole-cell patch electrophysiology to assess excitability more directly. Small (<25 μm in cell diameter) and medium-sized (25–35 μm) DRG neurons from both *LGI1*^fl/*Hoxb8*+^ and *LGI1*^fl/*Nav1.8*+^ mice showed no differences in their threshold for action potential firing (rheobase) compared with littermate controls or any difference in their passive electrophysiological properties (Fig. 3C, Supplementary Fig. 5I and Supplementary Table 4). However, unlike DRG neurons from *LGI1*^fl/*Nav1.8*+^ mice (Supplementary Fig. 5J and K), when measuring firing in response to prolonged current injections of increasing magnitude, small, but not medium DRG neurons from *LGI1*^fl/*Hoxb8*+^ mice fired significantly more action potentials around current threshold (between 150 and 250 pA) when compared with control neurons (Fig. 3D and E). To gain insight into the mechanisms governing this hyperexcitability, we assessed the impact of *LGI1* ablation on K_v_1 currents. K_v_1 channels contribute to *I*_KD_, a slowly inactivating voltage-dependent outward current.^36^ Focusing on small-diameter DRG neurons, total *I*_KD_ was significantly reduced in neurons from *LGI1*^fl/*Hoxb8*+^ mice versus controls (Fig. 3F and G). Dendrotoxin (DTX) is a specific blocker of K_v_1 channels (K_v_1.1, 1.2 and 1.6),^37^ and reduces total *I*_KD_ in neurons from *LGI1*^fl/*Hoxb8*−^ mice (Fig. 3F and G). The DTX-sensitive current is significantly smaller in *LGI1*-ablated neurons (Fig. 3H), suggestive of a loss of K_v_1 currents, which would explain the reduction in total *I*_KD_ (Fig. 3F and G).

### *LGI1* ablation leads to spinal hyperexcitability

**Figure 4:**
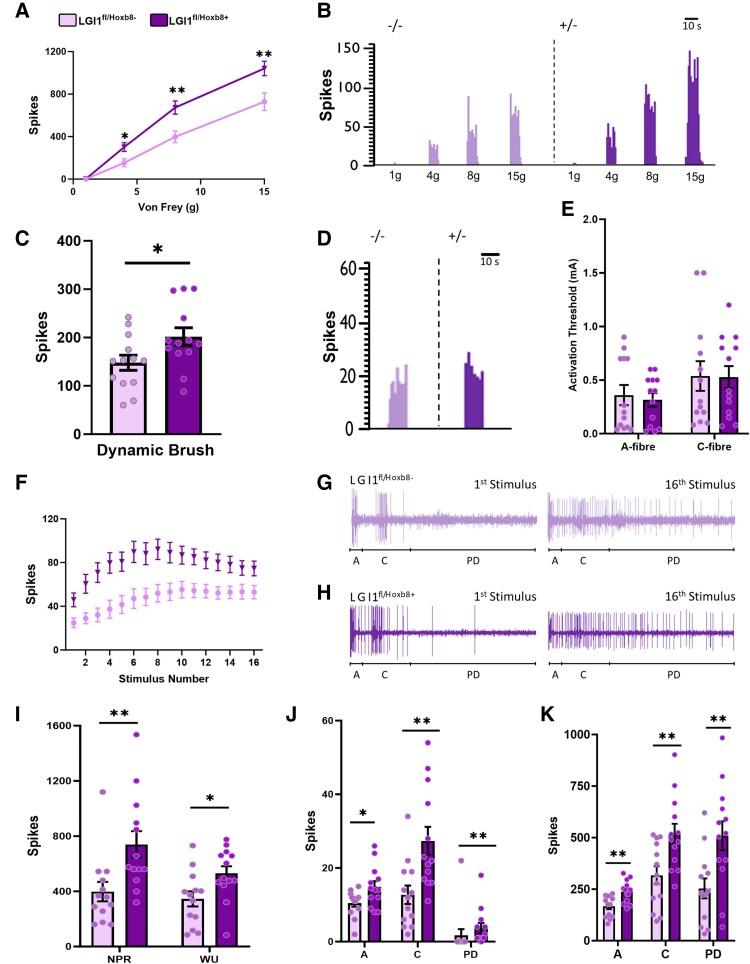
***LGI1* ablation leads to spinal hyperexcitability in *LGI1*^fl/*Hoxb8*+^ mice.** (**A**) Responses to punctate mechanical stimulation are enhanced in wide dynamic range (WDR) neurons from *LGI1*^fl/*Hoxb8*+^ mice compared with controls. (**B**) Representative histogram traces of single-unit responses to mechanical stimulation. (**C**) Responses to dynamic brushing are increased in WDR neurons from *LGI1*^fl/*Hoxb8*+^ mice compared with controls. (**D**) Representative histogram traces of single-unit responses to brush stimulation. (**E**) No differences were found between genotypes for electrical activation thresholds of WDR neurons in response to both A- and C-fibre input. (**F**) Wind-up curve plotted as total evoked spikes per stimulus. (**G** and **H**) Representative neurogram traces for *LGI1*^fl/*Hoxb8*−^ (**G**) and *LGI1*^fl/*Hoxb8*+^ (**H**) mice of single-unit responses, separated by latency for the first and last stimulus. (**I**) Total non-potentiated response (NPR) and wind-up (WU) are significantly increased in *LGI1*^fl/*Hoxb8*+^ mice compared with controls. (**J**) Neuronal events evoked by the first electrical stimulus separated by latency. Spikes during A- and C-fibre latencies and post discharge (PD) are increased in *LGI1*^fl/*Hoxb8*+^ mice. (**K**) Total neuronal events evoked by 16 electrical stimuli separated by latency. Spikes during A- and C-fibre latencies and during post discharge are increased in *LGI1*^fl/*Hoxb8*+^ mice. All data are shown as the mean ± SEM; *P < 0.05, **P < 0.01 and ***P < 0.001 versus *LGI1*^fl/*Hoxb8*−^ mice (*LGI1*^fl/*Hoxb8*−^, *n* = 13 mice; *LGI1*^fl/*Hoxb8*+^, *n* = 13 mice).

Next, we moved on to assess whether LGI1 might impact excitability at the level of the spinal cord. To do this, we performed *in vivo* extracellular recordings in *LGI1*^fl/*Hoxb8*+^ mice from WDR neurons located in the deep dorsal horn (recording depths: *LGI1*^fl/*Hoxb8*+^ 600 ± 19.4 μm; *LGI1*^fl/*Hoxb8*−^ 565 ± 43 μm). In line with behavioural mechanical hypersensitivity (Fig. 2A), we observed enhanced neuronal responses to Von Frey hair stimulation of the receptive field in *LGI1*^fl/*Hoxb8*+^ mice compared with *LGI1*^fl/*Hoxb8*−^ controls (Fig. 4A and B). We also observed a significantly increased response to dynamic brush stimulation following *LGI1* ablation (Fig. 4C and D). No differences were seen between genotypes in response to heat stimulation (Supplementary Fig. 6A and B), mirroring behavioural findings (Fig. 2B and C), and no differences were observed in response to noxious evaporative cooling following ethyl chloride application to the paw (Supplementary Fig. 6C and D). Similar thresholds for WDR neuron activation were observed between *LGI1*^fl/*Hoxb8*+^ and *LGI1*^fl/*Hoxb8*−^ mice for both A- and C-fibre input following electrical stimulation (Fig. 4E).

Next, we wanted to investigate whether LGI1 might play a role in pathological pain mechanisms within the spinal cord. Wind-up, the progressive increase in neuronal activity in response to a train of C-fibre input, is one of the neural mechanisms underlying central sensitization and can be measured in WDR neurons. Following a train of electrical stimulation (three times the C-fibre threshold) delivered to the receptive field, *LGI1*^fl/*Hoxb8*+^ displayed significantly enhanced wind-up (538.7 ± 50 spikes) compared with control mice (375.9 ± 51.4 spikes) (Fig. 4F–I). This wind-up response displayed a biphasic profile, with an accelerating phase preceding a plateau. *LGI1*^fl/*Hoxb8*+^ neurons displayed altered wind-up kinetics, with a faster rate in the accelerating phase [*LGI1*^fl/*Hoxb8*−^ rate constant (*k*) = 0.248; *LGI1*^fl/*Hoxb8*+^  *k* = 0.699, *P* < 0.05 Fig. 4F]. *LGI1*^fl/*Hoxb8*+^ mice also displayed an enhanced non-potentiated response, a measure of basal neuronal excitability (Fig. 4I). In response to the first electrical stimuli, WDR neurons from *LGI1*^fl/*Hoxb8*+^ mice showed increased action potential firing (spiking) to A- and C-fibre input compared with controls, in addition to post-discharge responses rarely seen in neurons from *LGI1*^fl/*Hoxb8*−^ mice (Fig. 4G, H and J). These same differences between genotypes were also observed when comparing total activity following the train of electrical stimulation (Fig. 4G, H and K).

*LGI1* ablation has been shown to cause the dysregulation of synaptic AMPA receptors.^15^ We therefore assessed the synaptic expression of AMPA receptors (GluA1 and GluA4) in lamina I and II of the SCDH from *LGI1*^fl/*Hoxb8*+^ mice. We looked at AMPA receptor expression at the postsynapse (co-localization with PSD95) and presynaptically (co-localization with synaptophysin), given the role of AMPA receptors in the presynaptic inhibition of primary afferents.^38^ However, we saw no difference in the expression of either GluA1 or GluA4 in the SCDH following *LGI1* ablation (Supplementary Fig. 7A–L). These results show that genetic ablation of *LGI1* from spinal neurons results in spinal hyperexcitability correlating with the enhanced pain-related sensitivity observed using behavioural assays, but with no gross changes in synaptic AMPA receptor expression.

### Genetic ablation of *LGI1* exacerbates nerve injury-induced pain

**Figure 5:**
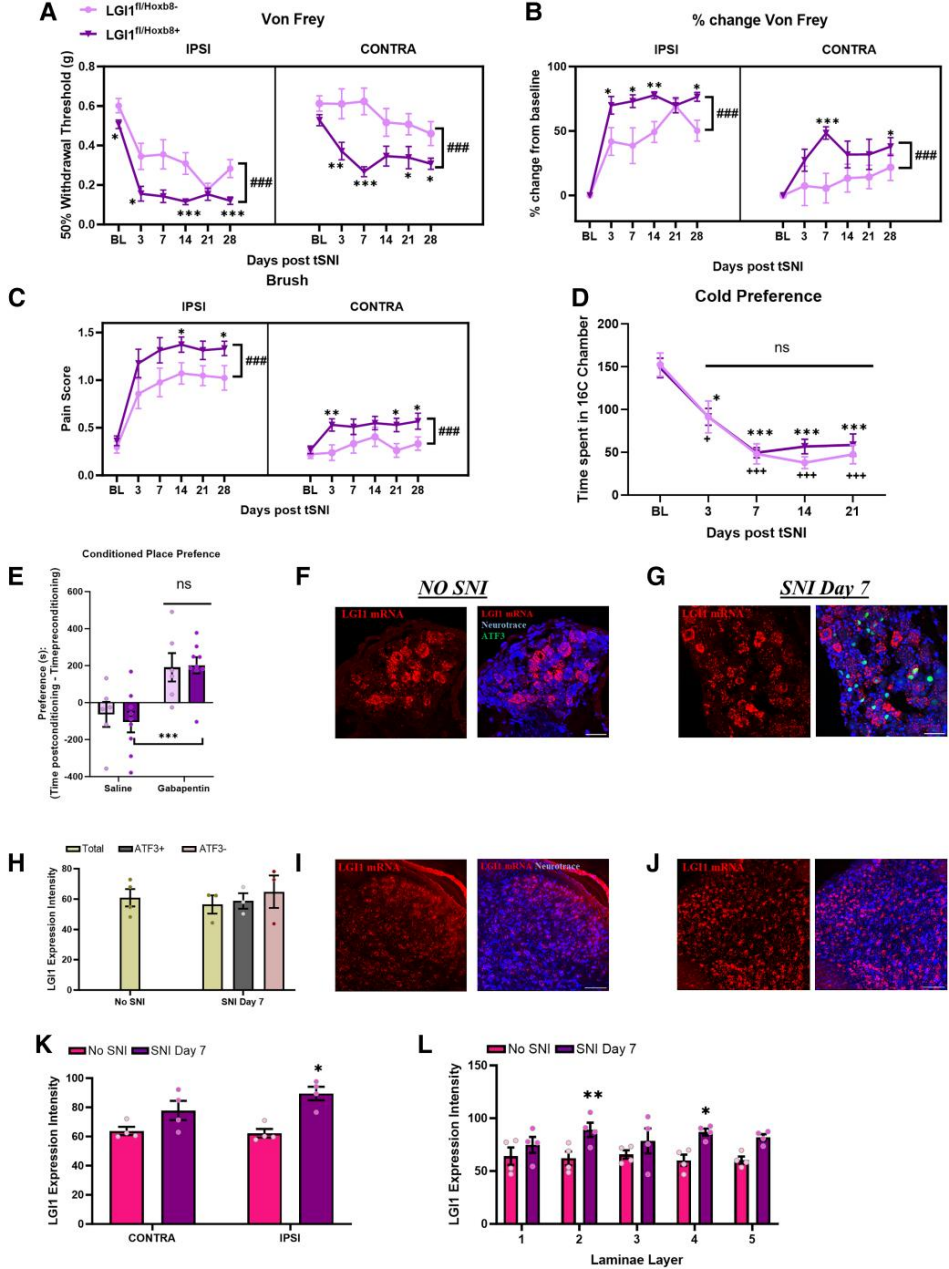
**Neuropathic pain is exacerbated in *LGI1*^fl/*Hoxb8*+^ mice.** (**A**) Both *LGI1*^fl/*Hoxb8*−^ (*n* = 14) and *LGI1*^fl/*Hoxb8*+^ (*n* = 17) mice developed ipsilateral mechanical hypersensitivity following nerve injury, as measured by Von Frey hair application. Ipsilateral mechanical hypersensitivity was significantly increased in *LGI1*^fl/*Hoxb8*+^ mice compared with controls. (**B**) Percentage increase in mechanical sensitivity, normalized to baseline, as measured by Von Frey hair application. (**C**) Both *LGI1*^fl/*Hoxb8*−^ and *LGI1*^fl/*Hoxb8*+^ mice developed ipsilateral mechanical allodynia following nerve injury, as measured by brush application. (**A**–**C**) *LGI1*^fl/*Hoxb8*+^ mice also developed significant contralateral pain hypersensitivity versus controls. (**D**) All mice developed cold hypersensitivity following nerve injury as measured by a decrease in the time spent at 16°C, but no differences were seen between genotypes. (**E**) Conditioned place preference test showing that on average mice from both genotypes developed a preference for the gabapentin paired chamber, indicating the development of spontaneous pain. However, no differences were seen between *LGI1*^fl/*Hoxb8*−^ (*n* = 6) and *LGI1*^fl/*Hoxb8*+^ (*n* = 8) mice. (**F** and **G**) Representative images of L4 mouse dorsal root ganglia (DRG) showing *in situ* hybridization (ISH) for *LGI1* mRNA (red) in naïve wild-type (WT) mice (**F**) or 7 days after nerve injury (**G**). Neurotrace (blue) was used to mark all neurons; ATF3 (green) shows injured neurons. (**H**) Quantification of *LGI1* mRNA signal in DRG neurons before (No SNI, *n* = 4) and 7 days after injury (*n* = 3), including both injured (ATF3+) and uninjured (ATF3−) neurons; scale bar = 50 µm. (**I** and **J**) Representative images of mouse L4 lumbar spinal cord showing ISH for *LGI1* mRNA (red) in naïve WT mice (**I**) or 7 days after nerve injury (**J**). Neurotrace (blue) was used to mark all neurons. (**K**) Quantification of *LGI1* mRNA signal in L4 spinal cord. An increase in expression was seen in the ipsilateral spinal cord 7 days after injury (*n* = 3) compared with naïve controls (*n* = 4). No significant increase was seen in the contralateral cord after injury. (**L**) Quantification of *LGI1* mRNA signal in L4 spinal cord before and 7 days after injury separated by laminae. All data are shown as the mean ± SEM. (**A**–**C**) *P < 0.05, **P < 0.01 and ***P < 0.001 versus *LGI1*^fl/*Hoxb8*−^ at specific time points; ^###^*P* < 0.001 versus *LGI1*^fl/*Hoxb8*−^. (**D**) *P < 0.05, ***P < 0.001, ^+^*P* < 0.05 and ^+++^*P* < 0.001 versus baseline (BL), ns (not significant) between genotypes. (**E**) ***P < 0.001 versus saline condition for all mice, ns between genotypes . (**K**) *P < 0.05 versus No spared nerve injury (SNI).

Central sensitization is a key component of neuropathic pain. Given our findings that *LGI1* ablation can enhance wind-up in dorsal horn neurons and the link to neuropathic pain in humans via the action of LGI1-Abs, we next wanted to investigate whether LGI1 had a role in regulating neuropathic pain sensitivity. To do this, we used the tibial spared nerve injury model of neuropathic pain, which induces robust pain-related hypersensitivity.^39^ Following nerve injury, both *LGI1*^fl/*Hoxb8*+^ and *LGI1*^fl/*Hoxb8*−^ mice developed mechanical pain hypersensitivity to Von Frey hair application versus baseline readings (Fig. 5A). Notably however, the degree of hypersensitivity was significantly exacerbated in *LGI1*^fl/*Hoxb8*+^ mice when compared with the control group and was evident throughout the 28-day testing period (with the exception of Day 21) (Fig. 5A). Furthermore, although mechanical sensitivity on the contralateral paw in control injured mice did not differ from baseline readings, *LGI1*^fl/*Hoxb8*+^ mice also developed a marked contralateral mechanical pain-related hypersensitivity up to Day 28, peaking at Day 7 (Fig. 5A). Given that withdrawal thresholds to Von Frey hairs are already reduced in uninjured *LGI1*^fl/*Hoxb8*+^ mice (see Fig. 2A and BL in Fig. 5A), we normalized responses to baseline. In agreement with the raw data, a greater percentage increase of mechanical pain-related hypersensitivity following nerve injury was seen in *LGI1*^fl/*Hoxb8*+^ mice compared with controls (Fig. 5B), confirming that loss of *LGI1* leads to the development of enhanced nerve injury-induced mechanical pain-related hypersensitivity, in addition to the manifestation of contralateral pain-related hypersensitivity (Fig. 5B).

We next assessed brush-evoked allodynia. Both *LGI1*^fl/*Hoxb8*+^ and *LGI1*^fl/*Hoxb8*−^ mice displayed nocifensive behaviours in response to brush stroke applied to the injured paw (Fig. 5C). As with the response to punctate mechanical stimuli, this response was also significantly exacerbated in *LGI1*^fl/*Hoxb8*+^ mice for ≤28 days (Fig. 5C). Remarkably, LGI1^fl/Hoxb8+^ mice also developed contralateral allodynia, albeit to a lesser extent to that seen in the ipsilateral paw (Fig. 5C).

The development of contralateral pain hypersensitivity was an unexpected finding and might have been influenced by stimulation of the injured paw.^40^ Therefore, we conducted studies where contralateral readings were taken without stimulation of the ipsilateral side and found that contralateral pain hypersensitivity following nerve injury in *LGI1*^fl/*Hoxb8*+^ mice was a robust result and not dependent on prior ipsilateral stimulation (Supplementary Fig. 8A–C).

Although all mice developed cold allodynia following nerve injury, there were no differences between genotypes (Fig. 5D). There were also no differences between groups in the degree of spontaneous pain behaviour exhibited 30–35 days after injury, as measured using the conditioned place preference paradigm (Fig. 5E). Although the exacerbation of nerve injury-induced mechanical hypersensitivity was clear when *LGI1* was removed from both DRG and spinal cord, no differences were observed in the development of neuropathic pain behaviour in *LGI1*^fl/*Nav1.8*+^ mice (Supplementary Fig. 8D–F). There was also no impact of sex on neuropathic pain exacerbation following *LGI1* ablation (Supplementary Tables 5 and 6).

To gain insight into how genetic removal of *LGI1* resulted in exacerbated pain in the tibial spared nerve injury model, we next assessed *LGI1* expression following peripheral nerve injury. Using ISH, at the level of the DRG no changes in *LGI1* mRNA were seen 7 days after tibial spared nerve injury in wild-type mice when compared with naïve control tissue (Fig. 5F–H), and levels remained normal in both injured and uninjured neurons (identified using the injury marker ATF3) (Fig. 5F–H). This lack of dysregulation is consistent with human data, where RNA-Seq showed no difference in *LGI1* expression between DRG taken from patients with and without neuropathic pain (Supplementary Fig. 2E; log fold change = 0.002, false discovery rate = 0.999). However, at the level of the spinal cord a significant increase in *LGI1* mRNA was seen in dorsal horn neurons 7 days post-injury in wild-type mice (Fig. 5I–K). This was particularly evident within lamina II and IV of the SCDH (Fig. 5L), with a trend towards increased expression in the contralateral cord (Fig. 5K).

In summary, loss of *LGI1* from both DRG and spinal cord neurons exacerbates mechanical pain-related hypersensitivity following nerve injury. LGI1 is upregulated in the dorsal horn following nerve injury, suggestive of a compensatory mechanism that is removed in *LGI1*^fl/*Hoxb8*+^ mice, resulting in the development of more severe sensory abnormalities. The regulation of allodynia, development of contralateral pain hypersensitivity and lack of exacerbation following specific ablation in Na_v_1.8+ sensory neurons point to a more central mechanism of LGI1 in regulating neuropathic pain.

## Discussion

Neuropathic pain remains a major clinical problem and is characterized by neuronal hyperexcitability at the level of the DRG and spinal cord. LGI1 is known to regulate excitability within the human nervous system and is the target of autoantibodies from neuropathic pain patients, but its role in regulating pain has not been studied directly. We find that LGI1 is highly expressed by DRG and SCDH neurons in both mouse and human. Using conditional genetic ablation in mice, we show, for the first time, a role for LGI1 in regulating pain sensitivity, with our data suggesting this to be mediated at the levels of both the DRG and the spinal cord. Loss of *LGI1* enhanced acute pain behaviours and increased DRG neuron excitability, with a reduction in K_v_1 currents. Pain behaviours were enhanced in the second phase of the formalin model in line with spinal hyperexcitability, including increased wind-up of dorsal horn neurons, and we also found that LGI1 regulated the severity of neuropathic pain.

The expression of LGI1 has been well characterized within the brain,^41,42^ given its association with the development of epilepsy and limbic encephalitis.^17,18^ Studies on its expression within the peripheral nervous system are lacking, despite the known presence of other members of the LGI family.^32^ Single-cell RNA-Seq of the mouse nervous system, however, indicates *LGI1* expression in DRG neurons, in particular nociceptors.^43^ In line with this, using ISH we found *LGI1* mRNA to be highly expressed in mouse DRG, albeit in both nociceptive and non-nociceptive neuron populations. LGI1 protein has been found in spinal cord extracts,^25^ and we showed its expression within SCDH neurons. Levels were comparable between inhibitory and putative excitatory neurons, with the lack of specificity for these broad populations similar to that seen in the hippocampus.^42^ This expression was translatable; we showed that LGI1 was also detected in bulk RNA-seq and spatial-sequencing data from human DRG, in addition to single-nuclear RNA-Seq from the human spinal cord. Importantly, we also assessed the expression of *ADAM11*, *ADAM22* and *ADAM23*, known interactors of LGI1.^12^ ADAM proteins are widely expressed within the nervous system,^44^ and we confirmed their expression within both DRG and SCDH neurons in mice and humans, demonstrating that, in addition to LGI1 itself, these regions also contain the substrates required for LGI1 activity.

We found that specific ablation of *LGI1* either in nociceptors or in all neurons of the caudal DRG and spinal cord resulted in subtle but significant hypersensitivity to acute pain stimuli. LGI1 interacts with K_v_1 channels to mediate the intrinsic excitability of neurons.^14^ At the level of the DRG, K_v_1 channels can act as a mechanosensitive brake in neurons, where blockade of K_v_1.1 results in mechanical pain-related hypersensitivity.^45^ To gain mechanistic insight, we assessed DRG neuron excitability and K_v_1 currents using whole-cell patch-clamp electrophysiology. Although genetic ablation of *LGI1* did not impact the action potential threshold, small-diameter DRG neurons from *LGI1*^fl/Hoxb8+^ mice displayed enhanced firing in response to prolonged graded current input. K_v_1 channels regulate repetitive firing in DRG neurons,^28,46^ and in line with this we found a reduction in K_v_1 currents. These observations are therefore likely to contribute to the development of mechanical pain-related hypersensitivity seen in *LGI1*^fl/*Hoxb8*+^ mice. We did not find similar changes in the excitability of small-diameter DRG neurons from *LGI1*^fl/*Nav1.8*+^ mice. LGI1 is a secreted protein that can act in a paracrine fashion to regulate the excitability of nearby neurons.^47-49^ We found high levels of *LGI1* mRNA in NF200+ (largely Na_v_1.8−) DRG neurons (Fig. 1). It is therefore possible that LGI1 released by Na_v_1.8− neurons could act on neighbouring small-diameter neurons to prevent K_v_1 channel disruption in *LGI1*^fl/*Nav1.8*+^ mice, in addition to mechanical pain hypersensitivity.

Given the expression of LGI1 in SCDH neurons and its role in synaptic transmission, we also undertook extracellular recordings of dorsal horn WDR neurons in *LGI1*^fl/*Hoxb8*+^ mice. These neurons are located in deeper spinal laminae (IV–VI), responding to both innocuous and noxious stimuli, with their firing rate being correlated with changes in stimulus intensity. As with DRG neurons, no changes in the activation threshold of WDR neurons were observed following *LGI1* ablation. However, consistent with our behavioural findings, we observed significantly enhanced neuronal responses to mechanical, but not thermal stimulation. WDR neurons are defined by their ability to respond to different sensory modalities. The selectivity of our findings for a specific modality (i.e. mechanical) suggests that, rather than altering the intrinsic properties of the WDR neuron (which would enhance responses to all modalities) in the non-potentiated spinal cord, the heightened mechanical sensitivity resulting from *LGI1* ablation has a presynaptic origin.

Previous studies have shown that genetic ablation of *LGI1* leads to enhanced excitatory synaptic transmission attributed to the presynaptic reduction of K_v_1 channels.^16,50,51^ It is plausible that the loss of K_v_1 currents we observed at the DRG neuron soma is also reflected at the central terminals of primary afferents, suggesting that disruption of presynaptic K_v_1 channels could also contribute to the enhanced spiking measured in SCDH neurons and the development of mechanical pain hypersensitivity.

We found that *LGI1*^fl/*Nav1.8*+^ mice displayed thermal hypersensitivity. Although removal of K_v_1 channels has also been linked to enhanced sensitivity to heat,^52^ this observation is difficult to explain owing to the lack of excitability changes in *LGI1*^fl/*Nav1.8*+^ DRG neurons and given that *LGI1* would have also been removed from *Nav1.8*+ neurons in *LGI1*^fl/*Hoxb8*+^ mice, in which no thermal hypersensitivity was observed.

We next used the formalin model to assess the role of LGI1 in situations of more tonic pain. We found that although genetic ablation of *LGI1* from nociceptors had no impact on pain-related behaviours in response to formalin, additional removal from spinal neurons (*LGI1*^fl/*Hoxb8*+^ mice) enhanced nociceptive behaviour in the second phase of this model, with this phase attributed to the sensitization of the spinal cord neurons.^34^ WDR neurons play a significant role in spinal plasticity. In the context of tissue or nerve injury, they have enhanced responses to noxious stimuli and display the phenomenon of wind-up, a short-term activity-driven plasticity, which can trigger neuronal hyperexcitability in line with heightened pain sensitivity in preclinical models,^53^ therefore making them highly relevant to our studies. In agreement with our behavioural findings, we showed that genetic ablation of *LGI1* from the spinal cord resulted in enhanced wind-up of WDR neurons.

Central sensitization, sharing features of wind-up, is a key aspect of neuropathic pain.^11^ In accordance, *LGI1*^fl/*Hoxb8*+^ mice displayed exacerbated mechanical hypersensitivity following nerve injury. This included increased sensitivity to both punctate stimulation and dynamic brush as a measure of mechanical allodynia. At the synaptic level, this potentiation of dorsal horn neurons results from the activation of C-fibres and the release of neuropeptides from central terminals along with glutamate. This leads to more prolonged depolarization, recruiting *N*-methyl-D-aspartate receptors, and together with AMPA receptor activation, increases intracellular calcium levels to amplify the response of the dorsal horn neuron.^54^ We found increased C-fibre input at the spinal level in *LGI1*^fl/*Hoxb8*+^ mice, which might have contributed to the increased wind-up of WDR neurons as shown previously.^55^

LGI1 is also expressed postsynaptically, where it has been shown to regulate AMPA receptor expression, hence function.^15,56^ Given that AMPA receptors contribute to central sensitization,^24^ we assessed their synaptic levels in the SCDH. We focused on two subunits: GluA1, because its dysregulation has been attributed to LGI1 loss,^16^ and GluA4, because changes in spinal synaptic expression are associated with pathological pain.^57^ In addition to postsynaptic levels, we also assessed presynaptic expression of AMPA receptors because they have been shown to contribute to presynaptic inhibition.^38^ When looking broadly across lamina I and II, we did not see any significant dysregulation of these subunits in comparison to control levels. Although previous studies are consistent in the development of hyperexcitability following *LGI1* ablation, they are conflicting on the underlying mechanisms. Some studies attribute findings to reduced K_v_1 currents, either by increasing intrinsic neuronal excitability or by enhancing synaptic transmission through loss of K_v_1 channels at the presynapse,^14,20,51^ although different groups suggest that the mechanism involves loss of AMPA receptors at the postsynapse^15,56^ and others suggest that both mechanisms might contribute.^16^ Given that AMPA receptors are excitatory channels, their loss is somewhat conflicting with the development of hyperexcitability, and therefore a reduction at the postsynaptic level of the WDR neuron would not account for the development of enhanced wind-up. However, a recent study provides a possible explanation, where LGI1 loss preferentially impacts AMPA receptors on inhibitory neurons, leading to an overall increase in network excitability.^58^

Although our findings in DRG neurons are consistent with a K_v_1 mechanism, we also observed that enhanced formalin and neuropathic pain behaviours (in which wind-up is a key mechanism) occurred only when *LGI1* was removed from spinal neurons, suggesting an additional role of spinal LGI1. Circuit-level modifications have been shown to contribute to the development of wind-up and pain hypersensitivity,^59,60^ and it is appreciated that neuropathic pain, including mechanical allodynia, results from the imbalance of spinal excitation and inhibition.^10,61-63^ Different populations of dorsal horn neurons have been shown to contribute to pain,^64^ and it is possible that our broad analysis of AMPA receptor expression might have missed synaptic changes occurring in specific subpopulations. Therefore, in addition to enhanced primary afferent input, loss of *LGI1* could induce circuit-level changes, similar to its role in seizure development, to enhance spinal mechanisms, including central sensitization, and exacerbate neuropathic pain. Consistent with its high expression in both regions, our findings point to both DRG and spinal actions of LGI1. The exact spinal mechanisms involved in the regulation of pain by LGI1 remain to be elucidated fully, and future studies specifically targeting spinal cord neurons will provide key insights.

To gain some understanding of why the genetic ablation of *LGI1* would increase neuropathic pain, we assessed its expression levels following nerve injury. We found no changes at the level of the DRG but, intriguingly, found that *LGI1* mRNA was increased in the spinal cord. This was contrary to our initial thoughts that LGI1 downregulation might contribute to neuropathic pain development. Instead, these findings point to a possible compensatory role of LGI1. Interestingly, K_v_1 channel expression can be regulated by neuronal activity as a homeostatic mechanism to maintain normal firing.^65^ It is therefore possible that LGI1 upregulation occurs in response to excessive spinal hyperexcitability to counteract these maladaptive changes, which are then lost in *LGI1*^fl/*Hoxb8*+^ mice, resulting in enhanced neuropathic pain. In agreement, compensatory upregulation of K_v_1 channels have been observed within primary afferents to limit the hyperexcitability induced by nerve injury, suggesting that LGI1 might represent a similar mechanism within the spinal cord.^66^

These findings also point to a possible therapeutic application. For example, increasing the availability of LGI1, either through application of recombinant protein or genetic expression, normalizes excitability,^14,49^ suggesting that enhancing this compensatory mechanism could limit spinal hyperexcitability and attenuate neuropathic pain. Surprisingly, we observed the development of contralateral pain hypersensitivity. Contralateral effects following unilateral nerve injury have been documented in animal models and shown to occur frequently in patients.^67-69^ Although the mechanisms are unclear, descending pathways from the brainstem, commissural spinal neurons and contralaterally projecting primary afferents have all been proposed to play a role.^40,69,70^ Given the exacerbation of ipsilateral pain, it is possible that that the altered ipsilateral input might have influenced contralateral readings.^40^ However, we found that contralateral pain hypersensitivity developed independent of ipsilateral stimulation. Although we did not look at LGI1 expression levels directly in the brainstem of *LGI1*^fl/*Hoxb8*+^ mice, these are unlikely to have been affected, given the expression profile of *Hoxb8*, which is restricted to caudal DRG and spinal cord.^27^ It is possible that the enhanced spinal input might have altered descending control or that more local spinal mechanisms play a role.^40,70^ Further investigation is needed to understand why loss of LGI1 would facilitate contralateral hypersensitivity, which might shed light on the mechanisms governing its development.

Neuropathic pain is a common feature of patients with antibodies targeting the voltage-gated potassium channel complex, which include antibodies against both LGI1 and CASPR2.^19,22^ Previous work has shown that CASPR2-Abs can cause pain by enhancing DRG neuron excitability via K_v_1 channel disruption.^28^ Neuropathic pain in LGI1-Ab patients is particularly responsive to immunotherapy,^22^ suggesting that these antibodies might also be causal to pain via disruption of their target. Our findings of enhanced excitability and increased pain sensitivity following LGI1 ablation support this notion. LGI1-Abs might target DRG neurons because they can bind to mouse primary sensory neurons *in vitro*.^22^ However, the level of binding is less than that seen for CASPR2-Abs, and although pain in CASPR2-Ab patients is typically length dependent (indictive of primary afferent involvement), it has a different, more truncal appearance in patients with LGI1-Abs.^22^ These observations are therefore suggestive of an additional locus of action beyond the DRG (e.g. the spinal cord), given our observations of spinal hyperexcitability following *LGI1* ablation in mice.

## Conclusion

In summary, we find that LGI1, the target of autoantibodies from neuropathic pain patients, plays a previously unrecognized role in regulating pain sensitivity. Disruption of LGI1 is known to increase excitability within the nervous system, although the underlying mechanisms are complex, including actions on intrinsic excitability, synaptic transmission (both pre and post) and circuit-level changes.^14,16,51,58^ Our findings while studying LGI1 involvement at the level of the DRG and spinal cord in relationship to pain point to a similar level of complexity, with overall agreement in the development of hyperexcitability following LGI1 disruption. This includes an action on DRG neurons, but also, in the pathological context, a role for spinal LGI1, given the observations of increased behaviour in the second phase of the formalin test, enhanced wind-up of spinal neurons, exacerbation of allodynia and the development of contralateral pain in spared nerve injury *LGI1*^fl/*Hoxb8*+^ mice, the increased expression of LGI1 in the spinal cord following nerve injury and the lack of enhanced formalin or neuropathic pain in *LGI1*^fl/*Nav1.8*+^ mice. Although future studies are needed to provide additional mechanistic insight, our findings implicate a novel action of LGI1 with relevance to clinical pain.

## Supplementary Material

awae302_Supplementary_Data

## Data Availability

All data and materials will be made available upon reasonable request to the corresponding author.

## References

[1] Van Hecke O, Austin SK, Khan RA, Smith BH, Torrance N. Neuropathic pain in the general population: A systematic review of epidemiological studies. Pain. 2014;155:654–662. 24291734 10.1016/j.pain.2013.11.013

[2] Baskozos G, Hébert HL, Pascal MMV, et al Epidemiology of neuropathic pain: An analysis of prevalence and associated factors in UK biobank. Pain Rep. 2023;8:e1066. 37090682 10.1097/PR9.0000000000001066 PMC7614463

[3] Fornasari D . Pharmacotherapy for neuropathic pain: A review. Pain Ther. 2017;6(Suppl 1):25–33. 29178034 10.1007/s40122-017-0091-4 PMC5701897

[4] Serra J, Bostock H, Solà R, et al Microneurographic identification of spontaneous activity in C-nociceptors in neuropathic pain states in humans and rats. Pain. 2012;153:42–55. 21993185 10.1016/j.pain.2011.08.015

[5] Djouhri L, Zeidan A, Alzoghaibi M, Al Otaibi MF, Abd El-Aleem SA. L5 spinal nerve axotomy induces distinct electrophysiological changes in axotomized L5- and adjacent L4-dorsal root ganglion neurons in rats *in vivo*. J Neurotrauma. 2021;38:330–341. 32993425 10.1089/neu.2020.7264

[6] Xiao Y, Wu Y, Zhao B, Xia Z. Decreased voltage-gated potassium currents in rat dorsal root ganglion neurons after chronic constriction injury. Neuroreport. 2016;27:104–109. 26671526 10.1097/WNR.0000000000000505

[7] Haroutounian S, Nikolajsen L, Bendtsen TF, et al Primary afferent input critical for maintaining spontaneous pain in peripheral neuropathy. Pain. 2014;155:1272–1279. 24704366 10.1016/j.pain.2014.03.022

[8] Woolf CJ . Central sensitization: Implications for the diagnosis and treatment of pain. Pain. 2011;152(Suppl.3):S2–S15. 20961685 10.1016/j.pain.2010.09.030 PMC3268359

[9] Todd AJ . Plasticity of inhibition in the spinal cord. Handb Exp Pharmacol. 2015;227:171–190. 25846619 10.1007/978-3-662-46450-2_9

[10] Peirs C, Williams SG, Zhao X, et al Mechanical allodynia circuitry in the dorsal horn is defined by the nature of the injury. Neuron. 2021;109:73–90.e7. 33181066 10.1016/j.neuron.2020.10.027 PMC7806207

[11] von Hehn CA, Baron R, Woolf CJ. Deconstructing the neuropathic pain phenotype to reveal neural mechanisms. Neuron. 2012;73:638–652. 22365541 10.1016/j.neuron.2012.02.008 PMC3319438

[12] Sagane K, Ishihama Y, Sugimoto H. LGI1 and LGI4 bind to ADAM22, ADAM23 and ADAM11. Int J Biol Sci. 2008;4:387–396. 18974846 10.7150/ijbs.4.387 PMC2575350

[13] Lancaster E, Burnor E, Zhang J, Lancaster E. ADAM23 is a negative regulator of Kv1.1/Kv1.4 potassium currents. Neurosci Lett. 2019;704:159–163. 30965109 10.1016/j.neulet.2019.04.012

[14] Seagar M, Russier M, Caillard O, et al LGI1 tunes intrinsic excitability by regulating the density of axonal Kv1 channels. Proc Natl Acad Sci U S A. 2017;114:7719–7724. 28673977 10.1073/pnas.1618656114 PMC5530646

[15] Fukata Y, Lovero KL, Iwanaga T, et al Disruption of LGI1-linked synaptic complex causes abnormal synaptic transmission and epilepsy. Proc Natl Acad Sci U S A. 2010;107:3799–3804. 20133599 10.1073/pnas.0914537107 PMC2840530

[16] Petit-Pedrol M, Sell J, Planagumà J, et al LGI1 antibodies alter Kv1.1 and AMPA receptors changing synaptic excitability, plasticity and memory. Brain. 2018;141:3144–3159. 30346486 10.1093/brain/awy253 PMC6202570

[17] Kalachikov S, Evgrafov O, Ross B, et al Mutations in *LGI1* cause autosomal-dominant partial epilepsy with auditory features. Nat Genet. 2002;30:335–341. 11810107 10.1038/ng832 PMC2606053

[18] Irani SR, Alexander S, Waters P, et al Antibodies to Kv1 potassium channel-complex proteins leucine-rich, glioma inactivated 1 protein and contactin-associated protein-2 in limbic encephalitis, Morvan’s syndrome and acquired neuromyotonia. Brain. 2010;133:2734–2748. 20663977 10.1093/brain/awq213 PMC2929337

[19] Gadoth A, Pittock SJ, Dubey D, et al Expanded phenotypes and outcomes among 256 LGI1/CASPR2-IgG positive patients. Ann Neurol. 2017;82(1): 79–92. 28628235 10.1002/ana.24979

[20] Schulte U, Thumfart JO, Klöcker N, et al The epilepsy-linked Lgi1 protein assembles into presynaptic Kv1 channels and inhibits inactivation by Kvβ1. Neuron. 2006;49:697–706. 16504945 10.1016/j.neuron.2006.01.033

[21] Extrémet J, El Far O, Ankri N, Irani SR, Debanne D, Russier M. An epitope-specific LGI1-autoantibody enhances neuronal excitability by modulating Kv1.1 channel. Cells. 2022;11:2713. 36078121 10.3390/cells11172713 PMC9454693

[22] Ramanathan S, Tseng M, Davies AJ, et al Leucine-rich glioma-inactivated 1 versus contactin-associated protein-like 2 antibody neuropathic pain: Clinical and biological comparisons. Ann Neurol. 2021;90:683–690. 34370313 10.1002/ana.26189 PMC8581990

[23] Tsantoulas C, McMahon SB. Opening paths to novel analgesics: The role of potassium channels in chronic pain. Trends Neurosci. 2014;37:146–158. 24461875 10.1016/j.tins.2013.12.002 PMC3945816

[24] Zhuo M . Ionotropic glutamate receptors contribute to pain transmission and chronic pain. Neuropharmacology. 2017;112(Pt A):228–234. 27543416 10.1016/j.neuropharm.2016.08.014

[25] Chabrol E, Navarro V, Provenzano G, et al Electroclinical characterization of epileptic seizures in leucine-rich, glioma-inactivated 1-deficient mice. Brain. 2010;133:2749–2762. 20659958 10.1093/brain/awq171 PMC2929330

[26] Nassar MA, Stirling LC, Forlani G, et al Nociceptor-specific gene deletion reveals a major role for Nav1.7 (PN1) in acute and inflammatory pain. Proc Natl Acad Sci U S A. 2004;101:12706–12711. 15314237 10.1073/pnas.0404915101 PMC515119

[27] Witschi R, Johansson T, Morscher G, Scheurer L, Deschamps J, Zeilhofer HU. Hoxb8-Cre mice: A tool for brain-sparing conditional gene deletion. Genesis. 2010;48:596–602. 20658520 10.1002/dvg.20656 PMC3566526

[28] Dawes JM, Weir GA, Middleton SJ, et al Immune or genetic-mediated disruption of CASPR2 causes pain hypersensitivity due to enhanced primary afferent excitability. Neuron. 2018;97:806–822.e10. 29429934 10.1016/j.neuron.2018.01.033 PMC6011627

[29] Watson C, Paxinos G, Kayalioglu G, Heise C. Chapter 16 - Atlas of the mouse spinal cord. In: Watson C, Paxinos G, Kayalioglu G, eds. The spinal cord. Academic Press; 2009:308–379.

[30] Tavares-Ferreira D, Shiers S, Ray PR, et al Spatial transcriptomics of dorsal root ganglia identifies molecular signatures of human nociceptors. Sci Transl Med. 2022;14:eabj8186. 35171654 10.1126/scitranslmed.abj8186 PMC9272153

[31] Yadav A, Matson KJE, Li L, et al A cellular taxonomy of the adult human spinal cord. Neuron. 2023;111:328–344.e7. 36731429 10.1016/j.neuron.2023.01.007 PMC10044516

[32] Kegel L, Aunin E, Meijer D, Bermingham JR. LGI proteins in the nervous system. ASN Neuro. 2013;5:167–181. 23713523 10.1042/AN20120095 PMC3691968

[33] McNamara CR, Mandel-Brehm J, Bautista DM, et al TRPA1 mediates formalin-induced pain. Proc Natl Acad Sci U S A. 2007;104:13525–13530. 17686976 10.1073/pnas.0705924104 PMC1941642

[34] Haley JE, Dickenson AH. Evidence for spinal N-methyl-D-aspartate receptor involvement in prolonged chemical nociception in the rat. Brain Res. 2016;1645:58–60. 26892026 10.1016/j.brainres.2016.02.001

[35] Mogil JS, Parisien M, Esfahani SJ, Diatchenko L. Sex differences in mechanisms of pain hypersensitivity. Neurosci Biobehav Rev. 2024;163:105749. 38838876 10.1016/j.neubiorev.2024.105749

[36] Madrid R, de la Pena E, Donovan-Rodriguez T, Belmonte C, Viana F. Variable threshold of trigeminal cold-thermosensitive neurons is determined by a balance between TRPM8 and Kv1 potassium channels. J Neurosci. 2009;29:3120–3131. 19279249 10.1523/JNEUROSCI.4778-08.2009 PMC6666436

[37] Harvey AL . Twenty years of dendrotoxins. Toxicon. 2001;39:15–26. 10936620 10.1016/s0041-0101(00)00162-8

[38] Lee CJ, Bardoni R, Tong CK, et al Functional expression of AMPA receptors on central terminals of rat dorsal root ganglion neurons and presynaptic inhibition of glutamate release. Neuron. 2002;35:135–146. 12123614 10.1016/s0896-6273(02)00729-8

[39] Shields SD, Eckert WA, Basbaum AI. Spared nerve injury model of neuropathic pain in the mouse: A behavioral and anatomic analysis. J Pain. 2003;4:465–470. 14622667 10.1067/s1526-5900(03)00781-8

[40] Luz LL, Lima S, Fernandes EC, et al Contralateral afferent input to lumbar Lamina I neurons as a neural substrate for mirror-image pain. J Neurosci. 2023;43:3245–3258. 36948583 10.1523/JNEUROSCI.1897-22.2023 PMC10162462

[41] Herranz-Pérez V, Olucha-Bordonau FE, Morante-Redolat JM, Pérez-Tur J. Regional distribution of the leucine-rich glioma inactivated (LGI) gene family transcripts in the adult mouse brain. Brain Res. 2010;1307:177–194. 19833108 10.1016/j.brainres.2009.10.013

[42] Ohkawa T, Fukata Y, Yamasaki M, et al Autoantibodies to epilepsy-related LGI1 in limbic encephalitis neutralize LGI1-ADAM22 interaction and reduce synaptic AMPA receptors. J Neurosci. 2013;33:18161–18174. 24227725 10.1523/JNEUROSCI.3506-13.2013 PMC3828467

[43] Zeisel A, Hochgerner H, Lönnerberg P, et al Molecular architecture of the mouse nervous system. Cell. 2018;174:999–1014.e22. 30096314 10.1016/j.cell.2018.06.021 PMC6086934

[44] Hsia HE, Tüshaus J, Brummer T, Zheng Y, Scilabra SD, Lichtenthaler SF. Functions of ‘A disintegrin and metalloproteases (ADAMs)’ in the mammalian nervous system. Cell Mol Life Sci. 2019;76:3055–3081. 31236626 10.1007/s00018-019-03173-7 PMC11105368

[45] Hao J, Padilla F, Dandonneau M, et al Kv1.1 channels act as mechanical brake in the senses of touch and pain. Neuron. 2013;77:899–914. 23473320 10.1016/j.neuron.2012.12.035

[46] Zhao X, Tang Z, Zhang H, et al A long noncoding RNA contributes to neuropathic pain by silencing Kcna2 in primary afferent neurons. Nat Neurosci. 2013;16:1024–1031. 23792947 10.1038/nn.3438 PMC3742386

[47] Lovero KL, Fukata Y, Granger AJ, Fukata M, Nicoll RA. The LGI1–ADAM22 protein complex directs synapse maturation through regulation of PSD-95 function. Proc Natl Acad Sci U S A. 2015;112:E4129–E4137. 26178195 10.1073/pnas.1511910112 PMC4522810

[48] Lugarà E, Kaushik R, Leite M, et al LGI1 downregulation increases neuronal circuit excitability. Epilepsia. 2020;61:2836–2846. 33104247 10.1111/epi.16736

[49] Extrémet J, Ramirez-Franco J, Fronzaroli-Molinieres L, et al Rescue of normal excitability in LGI1-deficient epileptic neurons. J Neurosci. 2023;43:8596–8606. 37863654 10.1523/JNEUROSCI.0701-23.2023 PMC10727174

[50] Lalic T, Pettingill Y, Vincent Y, Capogna M. Human limbic encephalitis serum enhances hippocampal mossy fiber-CA3 pyramidal cell synaptic transmission. Epilepsia. 2011;52:121–131. 21054347 10.1111/j.1528-1167.2010.02756.x

[51] Boillot M, Lee CY, Allene C, Leguern E, Baulac S, Rouach N. LGI1 acts presynaptically to regulate excitatory synaptic transmission during early postnatal development. Sci Rep. 2016;6:21769. 26878798 10.1038/srep21769 PMC4754946

[52] Clark JD, Tempel BL. Hyperalgesia in mice lacking the Kv1.1 potassium channel gene. Neurosci Lett. 1998;251:121–124. 9718989 10.1016/s0304-3940(98)00516-3

[53] McGaraughty S, Chu KL, Xu J. Characterization and pharmacological modulation of noci-responsive deep dorsal horn neurons across diverse rat models of pathological pain. J Neurophysiol. 2018;120:1893–1905. 30067136 10.1152/jn.00325.2018

[54] Latremoliere A, Woolf CJ. Central sensitization: A generator of pain hypersensitivity by central neural plasticity. J Pain. 2009;10:895–926. 19712899 10.1016/j.jpain.2009.06.012 PMC2750819

[55] Guan Y, Raja SN. Wide-dynamic-range neurons are heterogeneous in windup responsiveness to changes in stimulus intensity and isoflurane anesthesia level in mice. J Neurosci Res. 2010;88:2272–2283. 20209628 10.1002/jnr.22383

[56] Fukata Y, Adesnik H, Iwanaga T, Bredt DS, Nicoll RA, Fukata M. Epilepsy-related ligand/receptor complex LGI1 and ADAM22 regulate synaptic transmission. Science. 2006;313:1792–1795. 16990550 10.1126/science.1129947

[57] Cabañ Ero D, Baker A, Zhou S, et al Pain after discontinuation of morphine treatment is associated with synaptic increase of GluA4-containing AMPAR in the dorsal horn of the spinal cord. Neuropsychopharmacology. 2013;38:1472–1484. 23403695 10.1038/npp.2013.46 PMC3682142

[58] Fels E, Mayeur ME, Wayere E, et al Dysregulation of the hippocampal neuronal network by LGI1 auto-antibodies. PLoS One. 2022;17:e0272277. 35984846 10.1371/journal.pone.0272277 PMC9390894

[59] Hachisuka J, Omori Y, Chiang MC, Gold MS, Richard Koerber H, Ross SE. Wind-up in lamina I spinoparabrachial neurons: A role for reverberatory circuits. Pain. 2018;159:1484–1493. 29578943 10.1097/j.pain.0000000000001229 PMC6053328

[60] Fujiwara Y, Koga K, Nakamura NH, Maruo K, Tachibana T, Furue H. Optogenetic inhibition of spinal inhibitory neurons facilitates mechanical responses of spinal wide dynamic range neurons and causes mechanical hypersensitivity. Neuropharmacology. 2024;242:109763. 37852319 10.1016/j.neuropharm.2023.109763

[61] Tashima R, Koga K, Yoshikawa Y, et al A subset of spinal dorsal horn interneurons crucial for gating touch-evoked pain-like behavior. Proc Natl Acad Sci U S A. 2021;118:e2021220118. 33431693 10.1073/pnas.2021220118 PMC7826356

[62] Boyle KA, Gradwell MA, Yasaka T, et al Defining a spinal microcircuit that gates myelinated afferent input: Implications for tactile allodynia. Cell Rep. 2019;28:526–540.e6. 31291586 10.1016/j.celrep.2019.06.040 PMC6635381

[63] Peirs C, Williams SPG, Zhao X, et al Dorsal horn circuits for persistent mechanical pain. Neuron. 2015;87:797–812. 26291162 10.1016/j.neuron.2015.07.029 PMC4562334

[64] Todd AJ . Neuronal circuitry for pain processing in the dorsal horn. Nat Rev Neurosci. 2010;11:823–836. 21068766 10.1038/nrn2947 PMC3277941

[65] Zbili M, Rama S, Benitez MJ, et al Homeostatic regulation of axonal Kv1.1 channels accounts for both synaptic and intrinsic modifications in the hippocampal CA3 circuit. Proc Natl Acad Sci U S A. 2021;118:e2110601118. 34799447 10.1073/pnas.2110601118 PMC8617510

[66] Calvo M, Richards N, Schmid AB, et al Altered potassium channel distribution and composition in myelinated axons suppresses hyperexcitability following injury. Elife. 2016;5:e12661. 27033551 10.7554/eLife.12661 PMC4841771

[67] Arguis MJ, Perez J, Martínez G, Ubre M, Gomar C. Contralateral neuropathic pain following a surgical model of unilateral nerve injury in rats. Reg Anesth Pain Med. 2008;33:211–216. 18433671 10.1016/j.rapm.2007.12.003

[68] Konopka KH, Harbers M, Houghton A, et al Bilateral sensory abnormalities in patients with unilateral neuropathic pain; a quantitative sensory testing (QST) study. PLoS One. 2012;7:e37524. 22629414 10.1371/journal.pone.0037524 PMC3358252

[69] Enax-Krumova E, Attal N, Bouhassira D, et al Contralateral sensory and pain perception changes in patients with unilateral neuropathy. Neurology. 2021;97:E389–E402. 34011572 10.1212/WNL.0000000000012229

[70] Laflamme OD, Markin SN, Deska-Gauthier D, et al Distinct roles of spinal commissural interneurons in transmission of contralateral sensory information. Curr Biol. 2023;33:3452–3464.e4. 37531957 10.1016/j.cub.2023.07.014 PMC10528931

