# Supplementary material for: A role for leucine-rich, glioma inactivated 1 in regulating pain sensitivity
Source: Brain. 2024 Sep 20;148(3):1001–14. doi: 10.1093/brain/awae302 (PMC11884686; doi:10.1093/brain/awae302)
Supplement: awae302_Supplementary_Data [file awae302_supplementary_data.pdf]

Supplementary material

Supplementary Figures:

<u>Supp. Fig.1:</u>

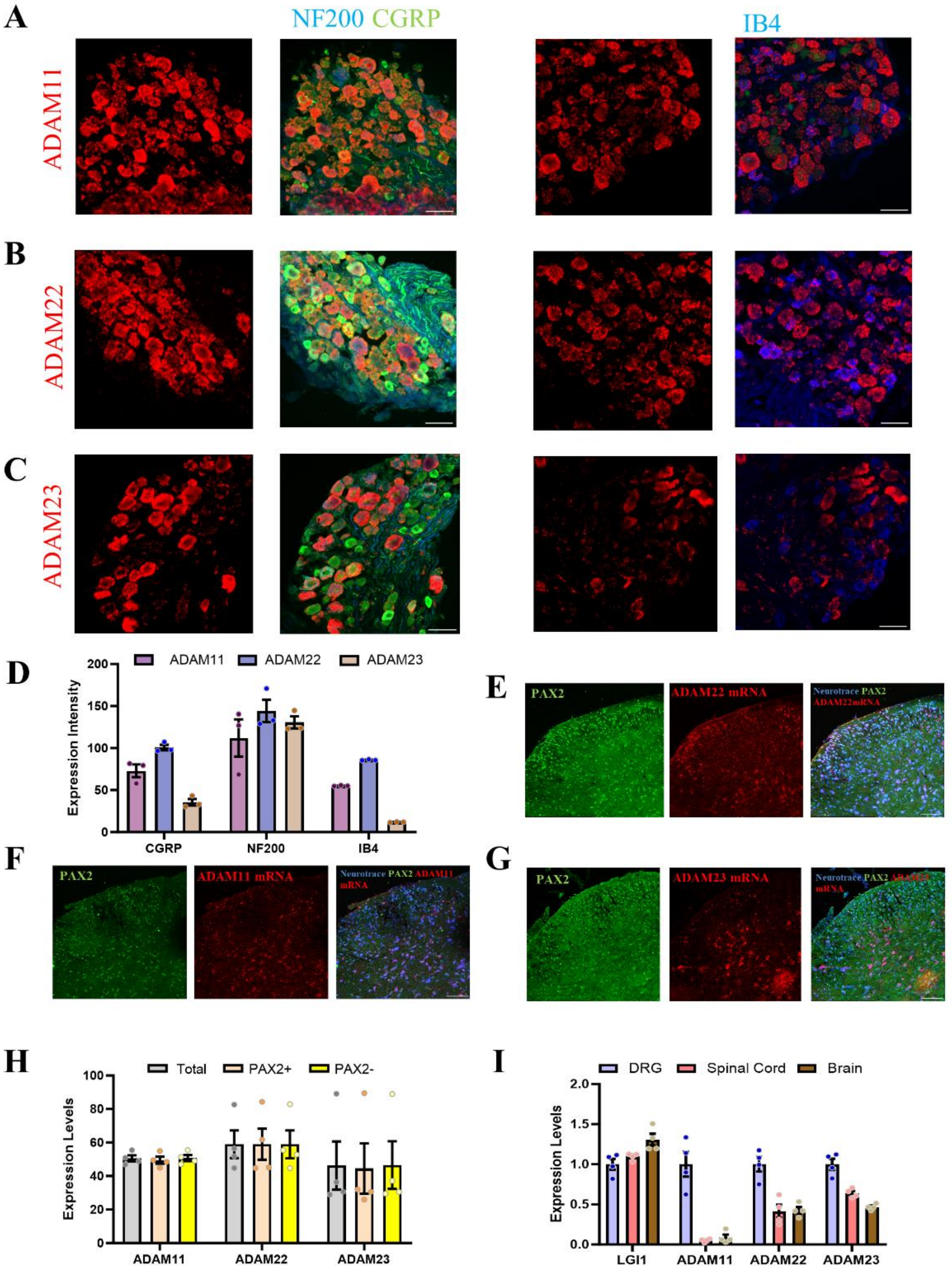

Supplementary Figure 1: Expression levels of *ADAM11*, *22* and *23* in DRG and spinal cord. (A-C) Representative images of mouse L4 DRG showing ISH for *ADAM11* **(A)**, *ADAM22* **(B)** and *ADAM23* **(C)** mRNA (red) in CRGP+ (green), IB4 (blue) and NF200+ (blue)

neurons, scale bar 50µm. **(D)** Quantification of *ADAM11*, *22* and *23* mRNA signal intensity in DRG neuron subtypes (n=3 mice). **(E-G)** Representative images of mouse lumbar spinal cord showing ISH for *ADAM11* **(E)**, *ADAM22* **(F)** and *ADAM23* **(G)** mRNA (red), with Neurotrace (blue) and Pax-2 (green) to mark inhibitory neurons, scale bar 100µm. **(H)** Quantification of *ADAM11*, *22* and *23* mRNA signal intensity in mouse lumbar spinal cord. (n=4 mice). **(I)** mRNA expression levels as measure by qPCR for *LGI1*, *ADAM11*, *22* and *23* from RNA extracted from whole mouse DRG, spinal cord and brain (n=4). Expression normalised to DRG levels. All data shown as mean±SEM.

Supp. Fig.2:

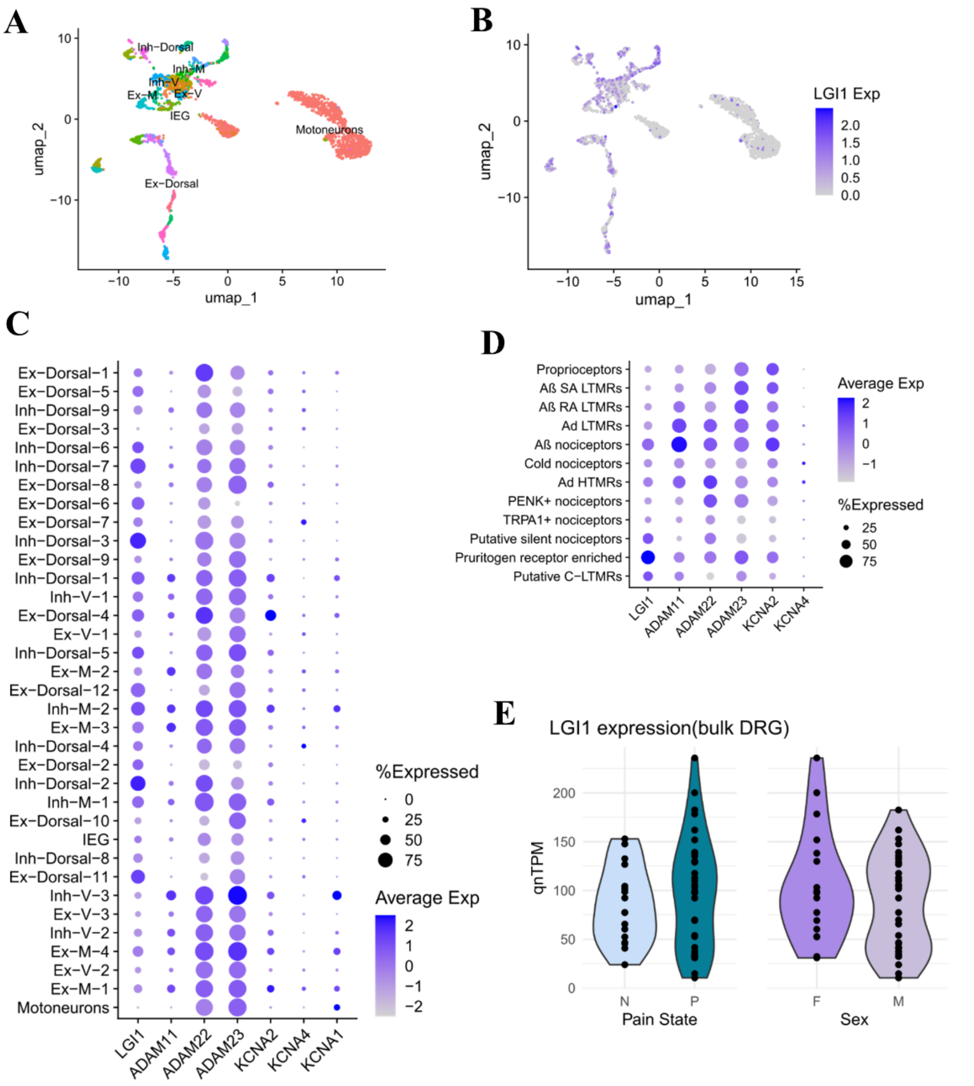

Supplementary Figure 2 *LGI1* and interaction partners are expressed in human DRG and spinal cord. (A, B) snRNA-Seq of human spinal cord. UMAP across spinal cord cell

types, presented with cell type labels **(A)** and *LGI1* expression **(B)**. **(C)** Dot plot summary of expression across all neuronal subtypes in human spina cord. **(D)** Dot plot highlighting human DRG expression from a published spatial-seq dataset. **(E)** Bulk RNA-seq expression of *LGI1* in hDRG between pain states (N = no pain, P = pain) and sex. qnTPM = quantile-normalized transcripts per million; LTMR = low threshold mechanoreceptor; HTMR = high threshold mechanoreceptor; IEG = immediate-early response gene subtype; Inh = inhibitory; Ex = excitatory. Exp = expression.

<u>Supp. Fig.3:</u>

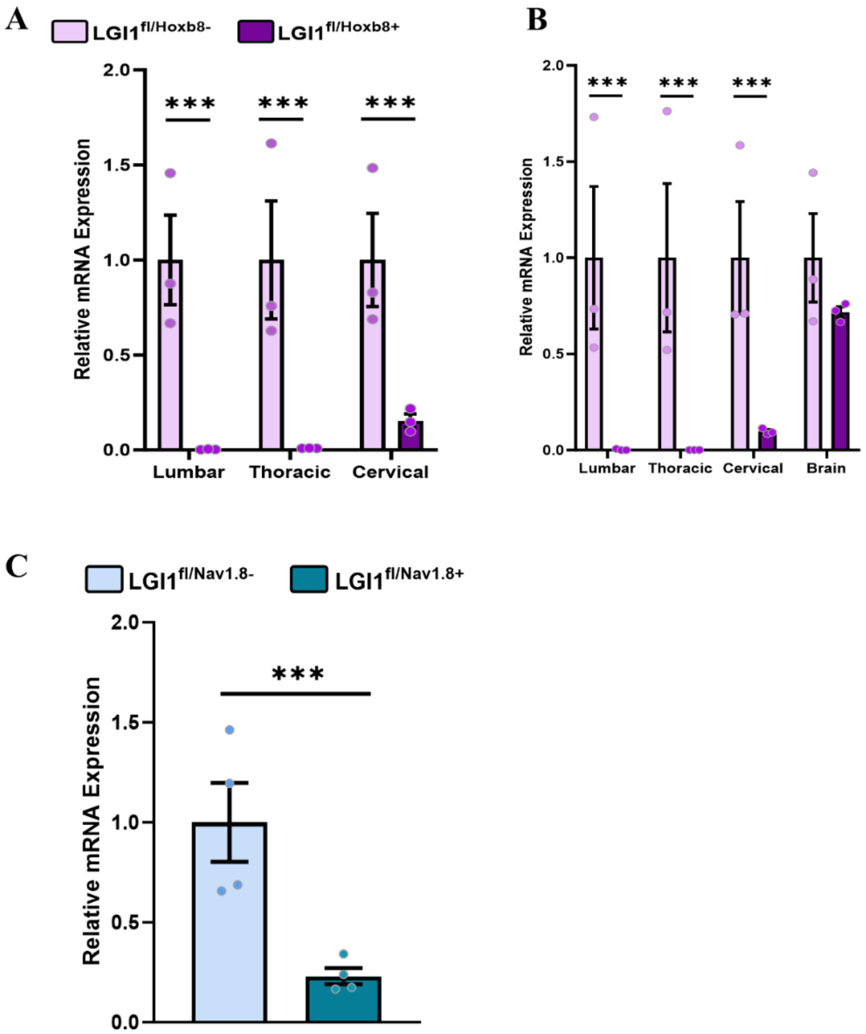

Supplementary Figure 3: *LGI1* expression in conditional KO mice (A, B) *LGI1* mRNA expression as measured by qPCR in *LGI1*$^{fl/Hoxb8+}$ (n=3) and *LGI1*$^{fl/Hoxb8-}$ mice (n=3). LGI1 expression is lost in lumbar and thoracic DRG **(A)** and spinal cord **(B)** in *LGI1*$^{fl/Hoxb8+}$ mice compared to controls. Levels of *LGI1* mRNA are significantly reduced in cervical DRG **(A)**

and spinal cord **(B)** of *LGI1*$^{fl/Hoxb8+}$ mice. *LGI1* mRNA is retained in the brain of *LGI1*$^{fl/Hoxb8+}$ mice **(B)**. **(C)** *LGI1* mRNA expression as measured by qPCR is significantly reduced in DRG from *LGI1*$^{fl/Nav1.8+}$ mice (n=4), compared to controls (*LGI1*$^{fl/Nav1.8-}$ mice, n=4). All data shown as mean±SEM, ***p<0.001 versus *LGI1*$^{fl/Hoxb8-}$ or *LGI1*$^{fl/Nav1.8-}$.

Supp. Fig.4:

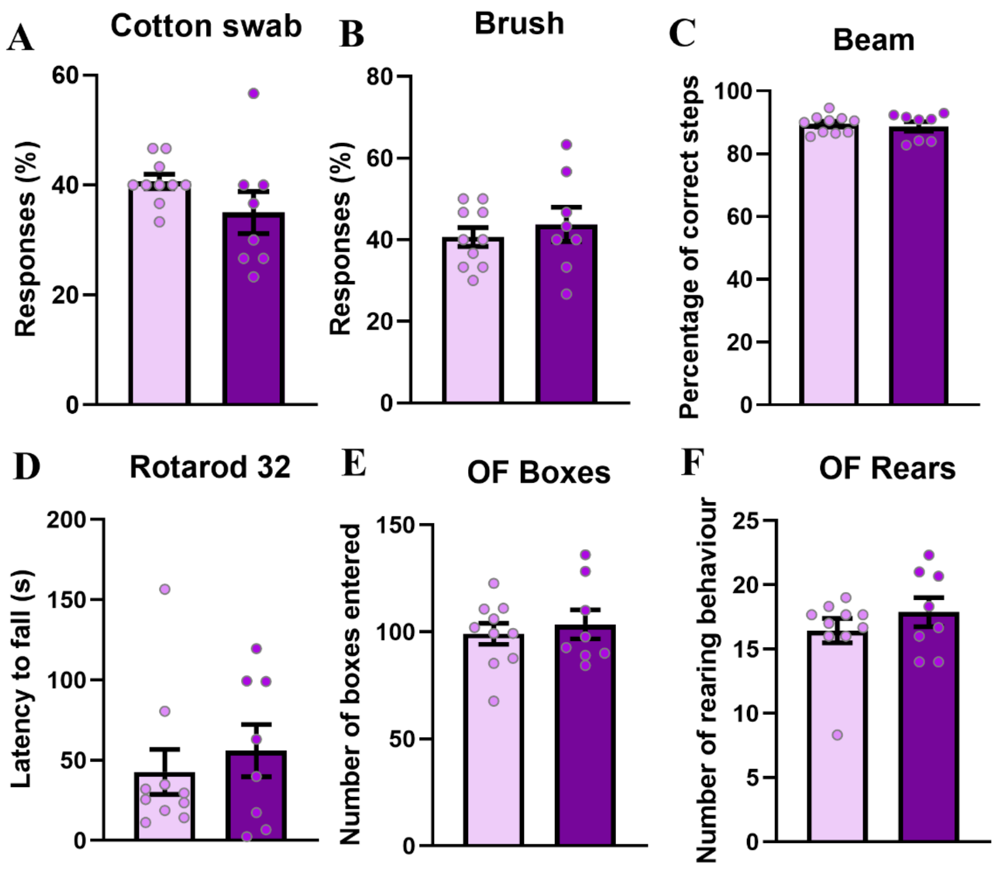

Supplementary Figure 4: Behavioural responses to innocuous mechanical stimuli and sensorimotor tasks in *LGI1*$^{fl/Hoxb8+}$ mice. (A, B) No difference between *LGI1*$^{fl/Hoxb8+}$ mice and littermate controls in the percentage response to either dynamic cotton swab (A) or brush (B) applied to the plantar surface of the hindpaw. (C, D) There is no difference between *LGI1*$^{fl/Hoxb8+}$ mice and controls on the beam test (percentage of correct steps) (C) or RotaRod (latency to fall) (D) when set at 32 rpm. (E, F) in the open field test, there was no difference in the number of boxes entered (E) or rearing behaviour (F) within a 3-minute period between genotypes. *LGI1*$^{fl/Hoxb8-}$ (n=10), *LGI1*$^{fl/Hoxb8+}$ (n=8). All data shown as mean±SEM

Supp. Fig.5:

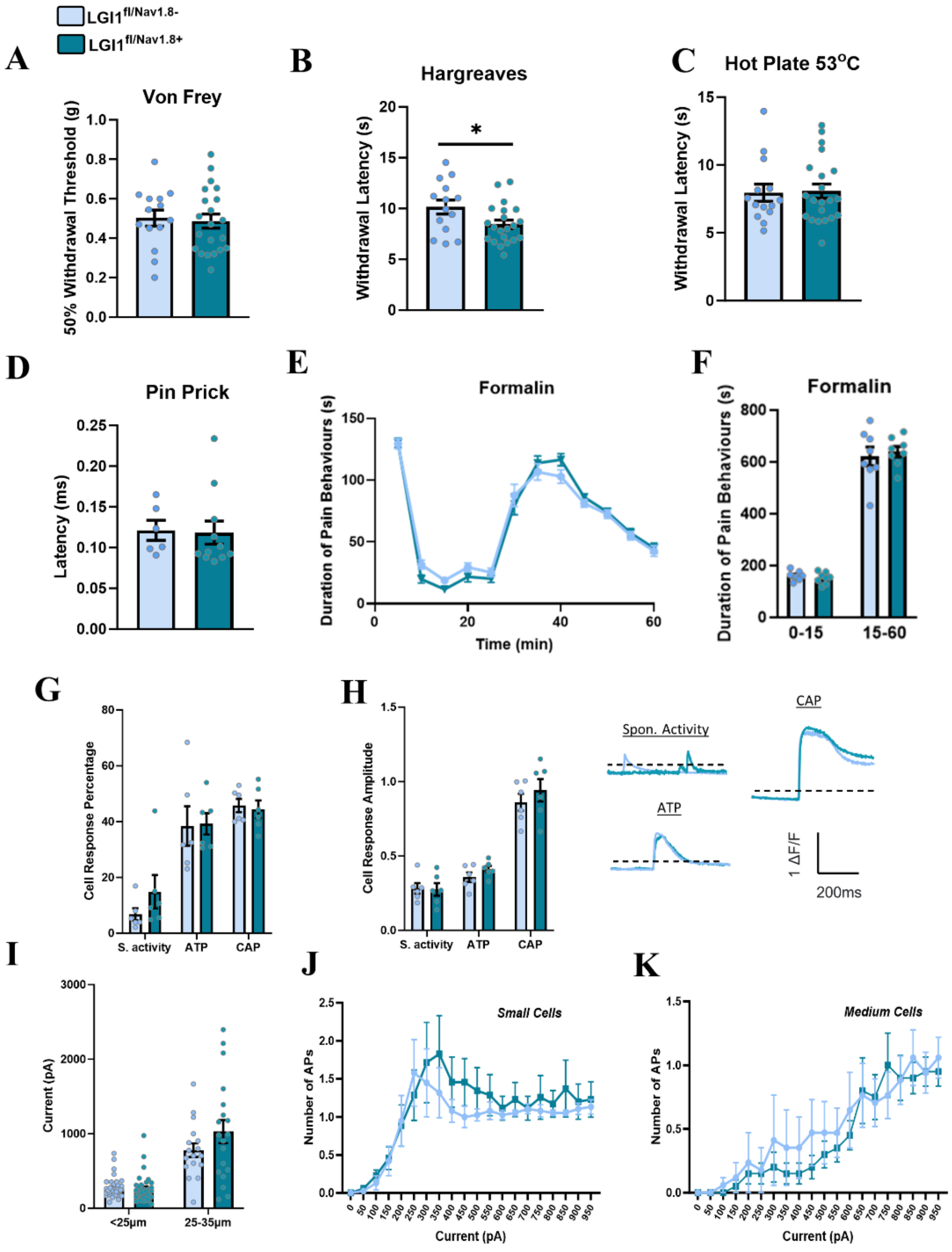

Supplementary Figure 5: Behavioural responses and DRG excitability measures in *LGI1*$^{fl/Nav1.8+}$ mice. (A) No differences between *LGI1*$^{fl/Nav1.8-}$ (n=14) and *LGI1*$^{fl/Nav1.8+}$ (n=21) mice in response to Von Frey hair application. **(B)** Decrease in pain-related heat thresholds in *LGI1*$^{fl/Nav1.8+}$ (n=21) mice on the Hargreaves test compared to littermate controls (n=14). **(C)** No differences in withdrawal latencies on the hot plate set at 53°C between *LGI1*$^{fl/Nav1.8-}$ (n=14) and *LGI1*$^{fl/Nav1.8+}$ (n=21) mice. **(D)** No differences in withdrawal latencies to pin prick

application between *LGI1*$^{fl/Nav1.8-}$ (n=6) and *LGI1*$^{fl/Nav1.8+}$ (n=11) mice. **(E)** *LGI1*$^{fl/Nav1.8+}$ mice (n= 8) display similar levels of nocifensive behaviour during the second phase of the formalin test when compared to littermate controls (n=8). **(F)** Total duration of nocifensive behaviour during for both the 1st phase (0-15mins) and 2nd phase (15-60mins) of the formalin test. **(G, H)** Calcium imaging data for DRG neurons cultured from *LGI1*$^{fl/Nav1.8-}$ and *LGI1*$^{fl/Nav1.8+}$ mice (n=6 mice, data taken from ~1900 cells per genotype) during the initial untreated period (spontaneous activity) and in response to both ATP (10 μM) and capsaicin (1μM). No difference between genotypes was seen for both the percentage of DRG neurons responding **(G)** or their amplitude of response **(H).** Neurons were identified by their response to 50mM KCL. **(H, right)** Example traces of calcium transients. **(I)** Whole cell patch clamp electrophysiology revealed no difference in the rheobase of both small (<25μm in cell diameter, n=35-39 cells taken from 4 mice) or medium (25-35μm, n=17-20 taken from 4 mice) sized DRG neurons between genotypes. **(J, K)** Action potential firing in response to a range of prolonged current injections in both small **(J)** and medium **(K)** sized DRG neurons**.** No differences were observed between genotypes. All data shown as mean±SEM. *p<0.05 versus *LGI1*$^{fl/Nav1.8-}$ mice.

Supp. Fig.6:

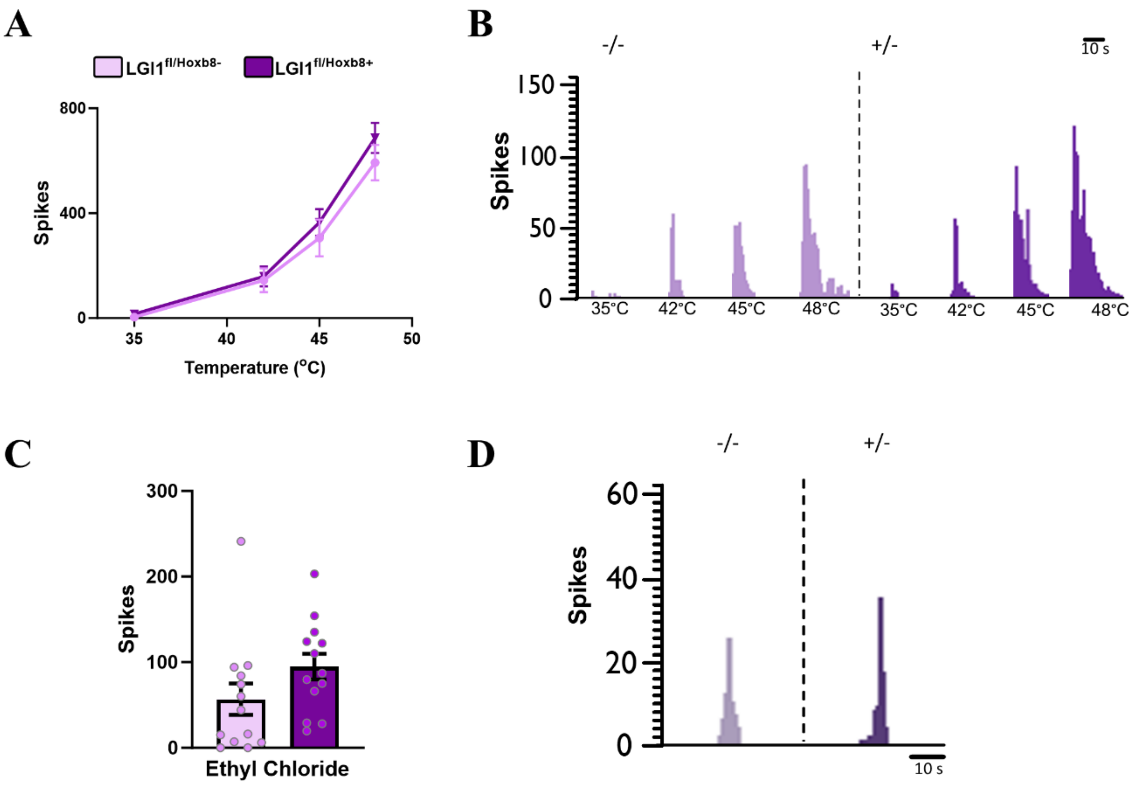

Supplementary Figure 6: Spinal excitability of *LGI1*$^{fl/Hoxb8+}$ WDR neurons to thermal stimulation. **(A)** No differences between genotypes in response to heat stimulation. **(B)** Representative histogram traces of single unit responses to heat in WDR neurons from both *LGI1*$^{fl/Hoxb8-}$ and *LGI1*$^{fl/Hoxb8+}$ mice. **(C)** No differences between genotypes in the response to noxious evaporative cooling induced by ethyl chloride application. **(D)** Representative histogram traces of single unit responses. All data shown as mean±SEM. (*LGI1*$^{fl/Hoxb8-}$, n=13 mice, *LGI1*$^{fl/Hoxb8+}$, n=13 mice).

Supp. Fig.7:

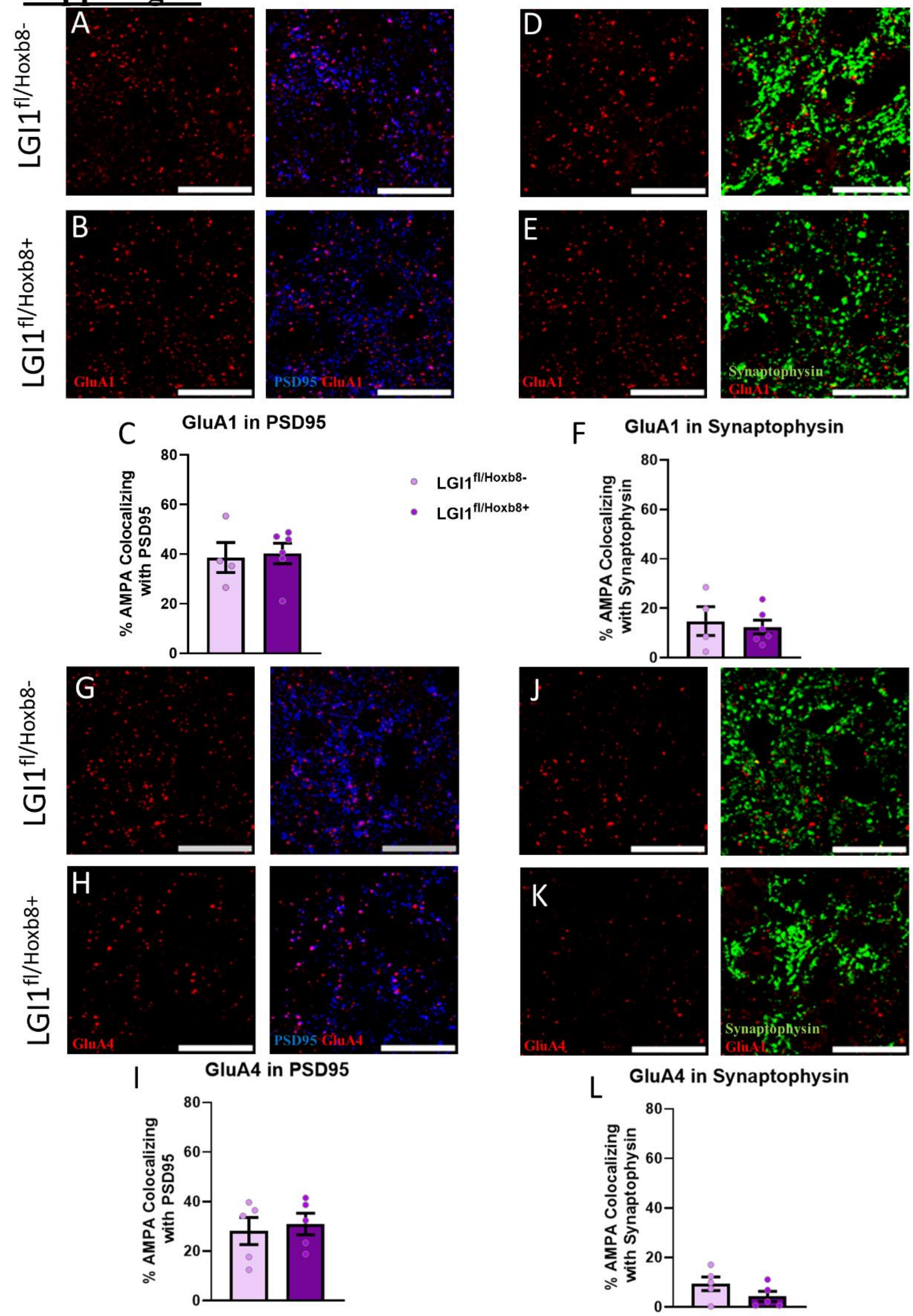

Supplementary Figure 7: Spinal synaptic AMPA receptor expression following *LGI1* ablation. (A, B) Representative images of GluA1 (red) and PSD95 (blue) immunoreactivity in lamina I+II of the lumbar SCDH from both *LGI1*$^{fl/Hoxb8-}$ **(A)** and *LGI1*$^{fl/Hoxb8+}$ **(B)** mice. **(C)**

Quantification of post-synaptic GluA1, as measured by the percentage of GluA1 colocalising with PSD95, shows no difference between genotypes. **(D, E)** Representative images of GluA1 (red) and synaptophysin (green) in lamina I+II of the lumbar SCDH from both *LGI1*$^{\text{fl}/\textit{Hoxb8-}}$ **(D)** and *LGI1*$^{\text{fl}/\textit{Hoxb8+}}$ **(E)** mice. **(F)** Quantification of pre-synaptic GluA1, as measured by the percentage of GluA1 colocalising with synaptophysin, shows no difference between genotypes. **(G, H)** Representative images of GluA4 (red) and PSD95 (blue) in lamina I+II of the lumbar SCDH from both *LGI1*$^{\text{fl}/\textit{Hoxb8-}}$ **(G)** and *LGI1*$^{\text{fl}/\textit{Hoxb8+}}$ **(H)** mice. **(I)** Quantification of colocalisation shows no difference between genotypes. **(J, K)** Representative images of GluA4 (red) and synaptophysin (green) in lamina I+II of the lumbar SCDH from both *LGI1*$^{\text{fl}/\textit{Hoxb8-}}$ **(J)** and *LGI1*$^{\text{fl}/\textit{Hoxb8+}}$ **(K)** mice. **(L)** Quantification of colocalisation shows no difference between genotypes. *LGI1*$^{\text{fl}/\textit{Hoxb8-}}$ n=5-6, *LGI1*$^{\text{fl}/\textit{Hoxb8+}}$ n=4-6. Scale bar 20μm.

Supp. Fig.8:

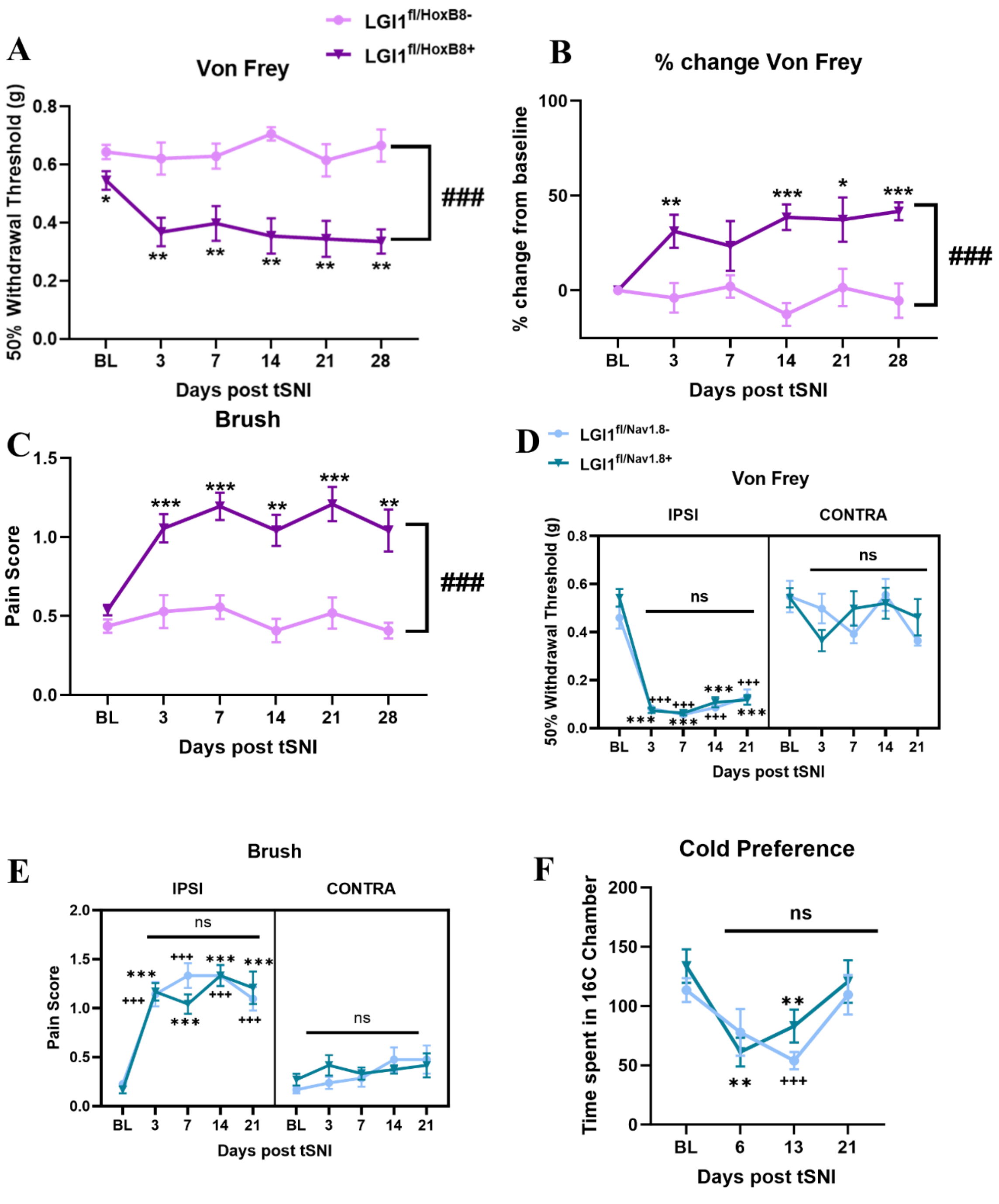

Supplementary Figure 8: Contralateral pain-related hypersensitivity in *LGI1*$^{fl/Hoxb8+}$ mice without ipsilateral stimulation and lack of neuropathic pain exacerbation in *LGI1*$^{fl/Nav1.8+}$ mice. (A) Signifcant development of contralateral pain hypersensitivity in *LGI1*$^{fl/Hoxb8+}$ mice (n=12) following SNI injury without ipsilateral pain testing compated to littermate controls (n=12). (B) Percentage increase in mechanical sensitivity, normalised to baseline, as measured

by Von Frey hair application (C) *LGI1*$^{fl/Hoxb8+}$ mice developed contralateral mechanical allodynia (as measures by response to brush application) following nerve injury, without ipsilateral stimulation. **(D)** Both *LGI1*$^{fl/Nav1.8-}$ (n=7) and *LGI1*$^{fl/Nav1.8+}$ (n=8) mice developed ipsilateral mechnical pain-related hypersensitivity following nerve injury, as measured by Von Frey hair application. There were no difference between genotypes and no development of contrlateral pain hypersensitivity. **(E)** There were no differences between genotypes in the development of mechnical allodynia following nerve injury as maeasured by brush stimulation. **(F)** There were no differences between genotypes in the development of cold pain hypersensitivity following nerve injury. All data shown as mean±SEM. $^{**}p<0.01$, $^{***}p<0.001$, $^{+++}p<0.001$ versus baseline.

Supplementary Tables:

Supplementary Table1: qPCR analysis of gene expression of *ADAMs 11, 22, 23* in DRG, spinal cord and brain of *LGI1*$^{fl/Hoxb8}$ mice.

Gene Name	**DRG cervical**		**DRG lumbar**		**DRG thoracic**	
	LGI1$^{fl/Hoxb8-}$	***LGI1***$^{fl/Hoxb8+}$	***LGI1***$^{fl/Hoxb8-}$	***LGI1***$^{fl/Hoxb8+}$	***LGI1***$^{fl/Hoxb8-}$	***LGI1***$^{fl/Hoxb8+}$
ADAM11	1±0.05	0.74±0.04*	1±0.14	0.57±0.13	1±0.12	1.33±0.31
ADAM22	1±0.01	0.87±0.01	1±0.01	0.97±0.07	1±0.03	1.09±0.07
ADAM23	1±0.06	0.89±0.05	1±0.09	1.01±0.15	1±0.05	0.89±0.02
LGI2	1±0.11	0.95±0.07	1±0.09	0.85±0.12	1±0.07	0.94±0.05
LGI3	1±0.09	0.98±0.09	1±0.13	0.92±0.14	1±0.06	1.03±0.06
LGI4	1±0.32	1.10±0.36	1±0.26	0.47±0.06	1±0.32	0.70±0.12

Gene name	**Spinal cord cervical**		**Spinal cord lumbar**		**Spinal cord thoracic**	
	LGI1$^{fl/Hoxb8-}$	***LGI1***$^{fl/Hoxb8+}$	***LGI1***$^{fl/Hoxb8-}$	***LGI1***$^{fl/Hoxb8+}$	***LGI1***$^{fl/Hoxb8-}$	***LGI1***$^{fl/Hoxb8+}$
ADAM11	1±0.02	0.85±0.03*	1±0.11	1.40±0.36	1±0.18	0.81±0.12
ADAM22	1±0.07	0.83±0.05	1±0.01	1.06±0.02	1±0.11	0.91±0.10
ADAM23	1±0.12	0.76±0.11	1±0.05	1.22±0.17	1±0.09	0.88±0.06
LGI2	1±0.08	0.84±0.09	1±0.03	1.10±0.12	1±0.12	0.96±0.09
LGI3	1±0.14	0.74±0.06	1±0.10	1.38±0.23	1±0.12	0.90±0.09
LGI4	1±0.64	0.29±0.09	1±0.34	0.92±0.21	1±0.12	0.89±0.09

Gene Name	**Brain**	
	LGI1$^{fl/Hoxb8-}$	***LGI1***$^{fl/Hoxb8+}$
ADAM11	1±0.12	1.64±0.54
ADAM22	1±0.05	1.04±0.09
ADAM23	1±0.07	1.11±0.17
LGI2	1±0.08	1.10±0.25
LGI3	1±0.11	1.08±0.14
LGI4	1±0.02	0.99±0.07

$^{*}p<0.05$ versus control, t test, n=4. Data shown as mean±SEM.

Supplementary Table 2: The effect of gender on behavioural outcomes in the *LGI1*$^{fl/Hoxb8}$ mouse line

Behaviour test (unit)	*LGI1*$^{fl/Hoxb8-}$		No. of mice			*LGI1*$^{fl/Hoxb8+}$		No. of mice			Effect of sex
	Female	Male	F	M	Total	F	M	F	M	Total	
**Pin Prick (ms)**	243±21.8	254±37.9	9	7	16	256±17.3	281±34.3	7	8	15	ns
**Hot Plate 53 (s)**	9.2±0.6	7.7±0.5	10	11	21	8.7±0.6	8.1±0.5	12	10	22	ns
**Von Frey (g)**	0.59 ±0.04	0.61±0.03	9	16	25	0.5±0.04	0.56±0.03	15	16	31	ns
**Hargreaves (s)**	10.3±0.5	9.7±0.5	4	15	19	10.1±0.4	9.3±0.5	10	12	22	ns
**Formalin 1st Phase (s)**	200.2±51.4	162.6±19.7	3	7	10	215.7±16.1	192.4±35.3	7	7	14	ns
**Formalin 2nd Phase (s)**	758.4 ±192.8	687.1±90.0	3	7	10	1004±58.2	828.9±55.6	7	7	14	ns
**Cotton swab (%)**	40±1.7	40±1.9	7	3	10	35.3±6.0	34.4±4	5	3	8	ns
**Brush (%)**	41.0±3.1	40±3.9	7	3	10	46.7±6.4	38.9±2.9	5	3	8	ns
**Open field (No. of Rears)**	17.1±0.4	14.8±3.2	7	3	10	18.9±1.6	16.1±1.3	5	3	8	ns
**Open field (No. of boxes)**	105.2 ±4.2	85.0±9.9	7	3	10	112.4±8.8	88.7±2.4	5	3	8	ns
**Rotarod (s)**	29.1±8.9	74.6±41.0	7	3	10	56.6±22.5	54.9±28.2	5	3	8	ns
**Beam (%)**	89.2±1.9	89.6±1.12	7	3	10	86.2±2.8	90.3±1.57	5	3	8	ns

Two Way-ANOVA with sex and genotype as factors, ns=not significant (p>0.05). F=female, M=male.

Supplementary Table 3: The effect of gender on behavioural outcomes in the *LGI1*$^{fl/Nav1.8}$ mouse line

Behaviour test (unit)	*LGI1*$^{fl/Nav1.8-}$		No. of mice			*LGI1*$^{fl/Nav1.8+}$		No. of mice			Effect of sex
	Female	Male	F	M	Total	F	M	F	M	Total	
**Pin Prick (ms)**	0.13±0.01	0.09	5	1 #	6	0.10±0.01	0.12±0.02	3	8	11	ns
**Hot Plate 53 (s)**	8.57±0.9	6.85±0.51	9	5	14	8.01±0.73	8.14±0.78	10	11	21	ns
**Von Frey (g)**	0.48±0.06	0.54±0.05	9	5	14	0.58±0.05	0.40±0.04	10	11	21	ns
**Hargreaves (s)**	11.32±0.79	8.01±0.61	9	5	14	9.40±0.66	7.58±0.39	10	11	21	ns
**Formalin 1st Phase (s)**	165.1±12.7	156±11.1	4	4	8	156±11.2	148.4±12.1	4	4	8	ns
**Formalin 2nd Phase (s)**	578.44 ±56.4	666.61 ±39.6	4	4	8	654.40 ±40	627.08 ±11.6	4	4	8	ns

Two Way-ANOVA with sex and genotype as factors, ns=not significant (p>0.05). F=female, M=male. # Sex effect not analysed due to low n number

Supplementary Table 4: Passive electrophysiological properties of cultured DRG neurons

	**Small (<25µm)**		**Medium (25-35 µm)**	
	LGI1 $^{fl/Nav1.8-}$	***LGI1*** $^{fl/Nav1.8+}$	***LGI1*** $^{fl/Nav1.8-}$	***LGI1*** $^{fl/Nav1.8+}$
**R Input (MΩ)**	284.81±16.25	301.07±25.15	109.49±8.38	96.88±19.93
**Capacitance (pF)**	14.37±0.57	14.16±0.70	24.92±1.34	27.04±1.33
**RMP (mV)**	-49.86±0.74	-49.86±0.91	-56.45±1.25	-57.76±1.10
**No. of cells**	39	35	17	20
	**Small (<25µm)**		**Medium (25-35 µm)**	
	LGI1 $^{fl/Hoxb8-}$	***LGI1*** $^{fl/Hoxb8+}$	***LGI1*** $^{fl/Hoxb8-}$	***LGI1*** $^{fl/Hoxb8+}$
**R Input (MΩ)**	270.88±15.80	285.29±19.33	86.69±9.71	65.22±5.86
**Capacitance (pF)**	14.91±0.53	15.11±0.51	29.55±1.03	30.67±1.04
**RMP (mV)**	-48.86±0.69	-49.17±0.66	-55.65±1.23	-57.24±0.85
**No. of cells**	47	46	23	23

No statistical differences in passive electrophysiological properties between *LGI1* ablated mice and littermate controls, p>0.05, t-test. Data shown as mean±SEM.

Supplementary Table 5: The effect of gender on nerve injury induced pain-related hypersensitivity in the *LGI1*$^{fl/Hoxb8}$ mouse line

Behaviour test (unit)	Time point (days) or group	***LGI1***$^{fl/Hoxb8-}$		No. of mice			***LGI1***$^{fl/Hoxb8+}$		No. of mice			Effect of sex
		**Female**	**Male**	**F**	**M**	**total**	**F**	**M**	**F**	**M**	**Total**	
**Von Frey – ipsilateral (g)**	0	0.57±0.07	0.61±0.05	4	10	14	0.52±0.03	0.5±0.03	7	10	17	ns
	3	0.26±0.16	0.36±0.08	4	10	14	0.09±0.03	0.2±0.06	7	10	17	ns
	7	0.23±0.1	0.39±0.1	4	10	14	0.16±0.06	0.13±0.03	7	10	17	ns
	14	0.16±0.04	0.36±0.07	4	10	14	0.13±0.03	0.11±0.02	7	10	17	ns
	21	0.15±0.05	0.19±0.04	4	10	14	0.11±0.03	0.19±0.05	7	10	17	ns
	28	0.2±0.06	0.31±0.06	4	10	14	0.12±0.02	0.12±0.03	7	10	17	ns
**Von Frey – contralateral (g)**	0	0.55±0.07	0.63±0.05	4	10	14	0.53±0.03	0.53±0.04	7	10	17	ns
	3	0.60±0.14	0.62±0.09	4	10	14	0.41±0.07	0.35±0.06	7	10	17	ns
	7	0.55±0.09	0.65±0.09	4	10	14	0.29±0.04	0.25±0.03	7	10	17	ns
	14	0.51±0.07	0.52±0.09	4	10	14	0.41±0.09	0.30±0.06	7	10	17	ns
	21	0.57±0.06	0.48±0.07	4	10	14	0.30±0.07	0.37±0.08	7	10	17	ns
	28	0.5±0.13	0.44±0.07	4	10	14	0.32±0.03	0.30±0.04	7	10	17	ns
**Brush – ipsilateral (pain score)**	0	0.45±0.11	0.23±0.07	4	10	14	0.41±0.09	0.33±0.06	7	10	17	ns
	3	1.2±0.29	0.73±0.19	4	10	14	1.5±0.14	1±0.21	7	10	17	ns
	7	1.3±0.24	0.83±0.18	4	10	14	1.5±0.18	1.2±0.19	7	10	17	ns
	14	1.3±0.25	1±0.13	4	10	14	1.4±0.14	1.3±0.1	7	10	17	ns
	21	1.1±0.21	1±0.13	4	10	14	1.4±0.11	1.3±0.15	7	10	17	ns
	28	1.1±0.21	1±0.17	4	10	14	1.4±0.09	1.3±0.11	7	10	17	ns
**Brush – contralateral (pain score)**	0	0.26±0.05	0.21±0.06	4	10	14	0.21±0.07	0.31±0.05	7	10	17	ns
	3	0.33±0.01	0.20±0.12	4	10	14	0.48±0.1	0.57±0.09	7	10	17	ns
	7	0.33±0.14	0.33±0.14	4	10	14	0.57±0.09	0.47±0.12	7	10	17	ns
	14	0.58±0.21	0.33±0.12	4	10	14	0.48±0.12	0.60±0.08	7	10	17	ns
	21	0.33±0.01	0.23±0.11	4	10	14	0.57±0.12	0.5±0.09	7	10	17	ns
	28	0.42±0.16	0.30±0.08	4	10	14	0.38±0.09	0.70±0.12	7	10	17	ns
**Cold Place preference**	0	165.5±13.1	139.2±29.4	3	3	6	165.3±2.9	114.9±32.5	4	5	9	ns
	6	**127.4±20.5***	**55.3±5.4**	3	3	6	94.8±10.6	88.7±16.5	4	5	9	*p<0.05 for *LGI1*$^{fl/Hoxb8-}$
	13	63.3±4.1	32.9±25.7	3	3	6	52.3±6.6	47.3±9.9	4	5	9	ns
	20	**51.6±4.1***	**24.1±7.4**	3	3	6	71.6±6.3	44.9±12.4	4	5	9	*p<0.05 for *LGI1*$^{fl/Hoxb8-}$
	27	51.4±16.8	43.7±21.2	3	3	6	56.5±17.8	60.1±20.1	4	5	9	ns
**Conditioned place preference**	Saline	-59.8±29.8	-68.2 ±147.9	3	3	6	-57±33.4	-143.9 ±97.6	4	4	8	ns
	Gaba-pentin	212.4 ±62.4	169.7 ±161.7	3	3	6	197.8 ±16.6	204.4 ±82.7	4	4	8	ns

Three Way repeated measures ANOVA with sex, genotype and time point or treatment as factors. ns=not significant (p>0.05). *p<0.05 female vs male for *LGI1*$^{fl/Hoxb8-}$ mice. F=female, M=male

Supplementary Table 6: The effect of gender on nerve injury induced pain-related hypersensitivity in the *LGI1*$^{fl/Nav1.8}$ mouse line

Behaviour test (unit)	Time point (days)	*LGI1*$^{fl/Nav1.8-}$		No. of mice			*LGI1*$^{fl/Nav1.8+}$		No. of mice			Effect of sex
		Female	Male	F	M	total	F	M	F	M	Total	
Von Frey – ipsilateral (g)	0	0.44±0.05	0.59	6	1#	7	0.55±0.05	0.54±0.05	5	3	8	ns
	3	0.08±0.01	0.09	6	1#	7	0.07±0.01	0.08±0.02	5	3	8	ns
	7	0.06±0.07	0.05	6	1#	7	0.06±0.01	0.06±0.02	5	3	8	ns
	14	0.09±0.01	0.08	6	1#	7	0.09±0.01	0.13±0.06	5	3	8	ns
	21	0.12±0.04	0.14	6	1#	7	0.1±0.01	0.15±0.04	5	3	8	ns
Von Frey – contralateral (g)	0	0.54±0.05	0.73	6	1#	7	0.53±0.06	0.056±0.06	5	3	8	ns
	3	0.45±0.05	0.63	6	1#	7	0.33±0.03	0.42±0.11	5	3	8	ns
	7	0.48±0.06	0.38	6	1#	7	0.59±0.09	0.35±0.07	5	3	8	ns
	14	0.52±0.06	0.61	6	1#	7	0.48±0.08	0.59±0.11	5	3	8	ns
	21	0.43±0.06	0.38	6	1#	7	0.54±0.11	0.34±0.05	5	3	8	ns
Brush – ipsilateral (pain score)	0	0.24±0.04	0.17	6	1#	7	0.18±0.06	0.14±0.03	5	3	8	ns
	3	1.12±0.14	1	6	1#	7	1.27±0.12	1±0	5	3	8	ns
	7	1.4±0.13	1	6	1#	7	1.13±0.13	0.89±0.11	5	3	8	ns
	14	1.33±0.12	1.33	6	1#	7	1.4±0.12	1.22±0.22	5	3	8	ns
	21	1.11±0.14	1	6	1#	7	1.33±0.24	1±0.19	5	3	8	ns
Brush – contralateral (pain score)	0	0.17±0.04	0.17	6	1#	7	0.27±0.06	0.28±0.15	5	3	8	ns
	3	0.22±0.07	0.33	6	1#	7	0.4±0.12	0.44±0.22	5	3	8	ns
	7	0.33±0.09	0	6	1#	7	0.27±0.07	0.44±0.11	5	3	8	ns
	14	0.55±0.11	0	6	1#	7	0.4±0.07	0.33±0	5	3	8	ns
	21	0.55±0.14	0	6	1#	7	0.4±0.12	0.44±0.29	5	3	8	ns

Three Way repeated measures ANOVA with sex, genotype and time point or treatment as factors. ns=not significant ($p>0.05$). F=female, M=male. # Sex effect not analysed due to low n number

Supplementary Table 7: Antibody list

**Antibody**	**Source**	**Identifier**
NeuN (1:500, Chicken)	Merck Millipore	Abn91
IB4(1:50, biotin conjugated)	Sigma-Aldrich	L2140
CGRP (1:250, Sheep)	Enzo	Ca1137
NF200 (1:1000, Rabbit)	Merck Millipore	ABN76
PAX2 (1:500, rabbit)	Thermo Fischer Scientific	71-6000
C-FOS (1:500, rabbit)	Synaptic Systems	226 008
ATF3 (1:500, rabbit)	Bio-Techne	NBP1-85816
GluA1 (1:300, rabbit)	Sigma-Aldrich	AB1504
GluA4 (1:300, rabbit)	Sigma-Aldrich	AB1508
PSD95 (1:200, guinea pig)	Nittobo Medical	Af660
Synaptophysin (1:1000, mouse)	Sigma-Aldrich	MABN1193
Neurotrace (1:500)	Thermo Fischer Scientific	N21482, N21479
Streptavidin Pacific Blue (1:100)	Thermo Fischer Scientific	Alexa Fluor (S11222)
Goat anti-rabbit Alexa Fluor 488 (1:500)	Thermo Fischer Scientific	Alexa Fluor (A110008)
Goat anti-chicken Alexa Fluor 546 (1:500)	Thermo Fischer Scientific	Alexa Fluor (A11040)
Donkey anti-sheep Alexa Fluor 488 (1:500)	Thermo Fischer Scientific	Alexa Fluor (A11015)
Donkey anti-mouse Alexa Fluor 488 (1:500)	Thermo Fischer Scientific	Alexa Fluor (A21202)
Donkey anti-rabbit Alexa Fluor 546 (1:500)	Thermo Fischer Scientific	Alexa Fluor (A10040)
Anti-Guinea Pig IgG (H+L), CF™405M (1:300)	Sigma-Aldrich	SAB4600467

Supplementary Table 8: Primers used for qPCR

Gene Name	Forward primer (5'-3')	Reverse primer (5'-3')
Lgi1	ATGACAACATTACAGGCACGTC	GGGCCACAATGACATAGAGC
Adam11	CCGCCCGGTCTTGGG	AGAGCCCTCTGGACTCTCTG
Adam22	AACTACCGAGAACAGAGACAGTT	TATAAAAAGAGTCGCCATCGCC
Adam23	CAGGCTGGGGATTTAAAAACG	ACGATTCAGATGGGGCCTTG
Lgi1-KO	TGCAAAGTCCCAAGACCTACC	TCTCTACGTGGTCCCATTCC
LGI2	CGTTCATCAGACTCTGCCGT	CGATGGACTGGCCTGTGATA
LGI3	CAGAGGTCATCTCCCTGACG	GGGCAGTGTCTGCAGGTTAT
LGI4	AGATCGAGAGGCATCCCACG	CATCCTGACGAGAGAGAGTGAC
18s	GGACCAGAGCGAAAGCATTTG	GCCAGTCGGCATCGTTTATG
Hprt1	GTCCTGTGGCCATCTGCCTAG	TGGGGACGCAGCAACTGACA
Gapdh	TGTGTCCGTCGTGGATCTGA	TTGCTGTTGAAGTCGCAGGAG

Supplementary methods:

Animal Care

All procedures were carried out in accordance with UK home office regulations and in line with the Animals Scientific Procedures Act 1986 at a licensed facility within the University of Oxford, following institutional review board approval. Animals were group housed in IVC cages in temperature and humidity-controlled rooms where food and water were available ad libitum, with a 12-hour light dark cycle. The welfare of all animals was continually assessed throughout all procedures. No animals were excluded from analysis. All experiments using mice are reported according to the ARRIVE guidelines.

Genotyping

Genotyping of offspring for transgenic mouse lines was performed by PCR of genomic DNA. For *LGI1*^{fl} mice primers (5'- ATTTCCTTAGTGCCCCTGTTTTTA- 3' to 5' – TGTCTGGATTCAATGCTGTCTTAGA- 3') were used to detect the presence of the floxed (150bp) or wild type allele (110bp). Primers (5' – AGCCTGTTTTGCACGTTCACC- 3' to 5' GGTTTCCCGCAGAACCTGAA- 3') were used to detect Cre recombinase in *LGI1*$^{fl/Nav1.8+/-}$ and *LGI1*$^{fl/Hoxb8+/-}$ mice. No band indicated a Cre negative mouse, with an internal control (5'- CCTAGCACCCACCCAAAGAGCTG-3' to 5'- GGTCCTCACTGGCAGCAGCTGCA-3') used to confirm DNA amplification.

Behavioural tests

Von Frey

Mechanical sensitivity was assessed by placing mice in a Perspex box situated on top of a wire mesh. Calibrated Von Frey hairs (Ugo Basile) were applied to the plantar surface of the hind paw and a reflex withdrawal response used to calculate the 50% withdrawal threshold as shown previously.[1]

Brush

Dynamic mechanical allodynia was assessed using a small modified paintbrush (5/0, The Art Shop) to stroke the plantar surface of the paw.[2] A scoring system was used to determine a dynamic allodynia score, (0) (a nonpainful response) lifting of the paw for less than 1 s, (1) sustained lifting of the paw or a single flinch, (2) lateral paw lift above the level of the body or a startle like jump and (3) multiple flinching responses or licking of the affected paw.

Pin Prick

The response to pin prick was assessed as previously described.[3] A dissecting pin was attached to a 1g Von Frey filament and applied to the plantar surface of the hind paw to elicit a rapid withdrawal reflex. The latency to withdraw was recorded using GoPro at 240 fps and analyzed using the video editing program Avidemux. Three measurements were taken for each hind paw and the averaged latency to withdraw was measured.

Hargreaves

Thermal sensitivity was assessed using the Hargreaves method.[4] Using the Hargreaves apparatus (Ugo Baslie) a radiant heat source was applied to the plantar surface of the hind paw and the latency to withdrawal was used to determine heat sensitivity threshold. Three measurements were taken for each hind paw and the averaged latency to withdraw was measured.

Hot Plate

Response to a suprathreshold heat stimulus was measured using the hot plate (Ugo Basile) assay. A metallic plate was set at 53°C. Mice were chosen at random from their home cage and placed onto the plate and the latency until a response, in this case shaking, licking, or biting of the paw, was measured.

Cold Preference test

To assess cold sensitivity a thermal preference paradigm was used. The thermal preference equipment (Ugo Basile) consisted of two plates with a small connecting bridge. The plates were set at either 16°C or room temperature. Mice were chosen at random from their home cage and assessed over a 10-minute period and the percentage of time spent at 16°C was calculated.

Formalin test

Mice received an intraplantar injection of 20 µl of 5% formalin diluted from formaldehyde solution (5% v/v from 37% stock formaldehyde solution (Sigma)) in sterile saline. Mice were placed in a Perspex cylinder and video recorded over a 60-minute period. The duration of pain-related behaviour, biting/licking/paw lifting, was measured for each 5-minute period. recorded over a 60-minute period. Data was subsequently pooled for the first (0-15mins) and second (15-60mins) phases. Spinal tissue was collected 2 hours post formalin injection for c-Fos analysis.

Conditioned Place Preference

To test for ongoing pain-related behaviour, a three-day conditioning protocol using a biased chamber assignment was performed. The custom 3-chamber apparatus consisted of two conditioning side chambers connected by a centre chamber (three chambered San Diego Instruments - dimensions of side chamber: 17cm W x 15cm D x 20cm H; dimensions of central chamber: 7cm W x 15cm D x 20cm H. Plexiglass inserts x2 for each central chamber – external dimensions: 6.5cm W x 2.5cm D x 17.5cm H). Isolation chamber (San Diego Instruments) – external dimensions: 60cm W x 29.5cm D x 50.5cm H). Mice were able to discriminate between chambers using visual (vertical black-and-white striped walls versus black-and-white spot walls) and sensory (strawberry versus vanilla scent) cues. On day 1 (acclimatisation, 4-5 weeks after SNI surgery), mice had free access to explore all chambers for 30 minutes. On days 2 and 3 (preconditioning), mice were again allowed to freely explore for 30 min whilst their position was recorded using an infrared camera and AnyMaze 7.16 software (Stoelting, USA). To avoid pre-existing chamber bias, mice spending more than 80% or less than 5% of time in either side chamber during preconditioning were excluded. For conditioning (days 4 to 6), mice received an i.p. vehicle injection (saline). They were returned to their home cage for 5 min, then confined to their preferred side chamber for 30 min. Four hours later, mice received an i.p. injection of gabapentin (100 mg/kg)[5]. They were returned to their home cage for 5 min,

and then placed in their non-preferred chamber for 30 min. On test day (day 7), mice could freely explore all chambers whilst their position was recorded, as during pre-conditioning, for 30 min. Difference scores were calculated as the time spent in each chamber on test day minus the mean time spent during pre-conditioning.

Rotarod

Mice were placed on a rotating rod set at a constant speed of 32 rpm. The latency until the mouse could no longer stay on the rod was recorded and the average taken from 3 separate trials. A cut off of 3 minutes was used.

Open Field test

A black box exhibiting a grid system of 3x4 squares outlined on the floor was used. Mice were placed in this box for 3 minutes and the number of squares the mouse entered during this period was recorded, as well as the number of rears (standing on the hindlegs in an upright posture). Separate trials were performed and an average taken.

Bean walk test

A wooden beam of about 1 m in length, elevated from the bench surface was used. Mice were video recorded as they moved along the beam and the percentage of correct steps was calculated by counting the number of missed steps and comparing to the total number of steps taken for each of 3 runs.

Brush and cotton swab

The plantar hind paws of mice were brushed with a fine artists paint brush or a cotton swab that had been puffed out to 3 times its original size. Each mouse received 5 successive stimuli on alternate hind paws (10s apart), twice. The number of responses were recorded in which a response included, lifting, moving the hind paw or walking away from the stimulus. Mice were tested on 3 different days to obtain an average baseline value.

Spared Nerve injury

Mice were anesthetized using isoflurane inhalation and the left sciatic nerve and peripheral branches: common peroneal, tibial and sural nerves were exposed. The common peroneal and sural nerves were ligated, leaving the tibial nerve intact. The ligated nerves were transected distally, and a 2 mm section removed to prevent nerve regeneration. The muscle and skin

incision were stitched and post-operative care and analgesics were given (a single local injection into the muscle of 2 mg/kg Marcain, AstraZeneca and a single subcutaneous injection of 5 mg/kg Rimadyl, Pfizer).

Cell culture

Adult male and female mice of 4-8 weeks of age were sacrificed in a CO2 chamber. The spinal column was rapidly removed and bisected to reveal the DRG. DRG were taken from all levels, placed directly into Hanks' Balanced Salt solution (HBSS without Ca^{2+} and Mg^{2+}, Invitrogen) and subjected to enzymatic digestion using collagenase II (12mg/ml, Worthington) and dispase II (14mg/ml, Roche) diluted in HBSS for 1.5 hours at 37°C. DRGs were then mechanically dissociated using fire polished pipettes and dissociated cells suspended in culture medium (Neurobasal medium, 2% B27, 1% GlutaMAX™, GIBCO, 1% antibiotic/antimycotic (ThermoFisher Scientific)) supplemented with mouse NGF (50ng/ml, Peprotech) and GDNF (10ng/ml, Peprotech), and plated on to 13 mm coverslips precoated with laminin (R&D Systems) and poly-D Lysine (BD biosciences) before being incubated at 37°C.

Calcium imaging

Coverslips were incubated for 45-90 min at 37°C with 1 μM Fura-2AM (Invitrogen) in Neurobasal medium supplemented as above. After incubation, coverslips were transferred to artificial extracellular fluid (ECF; 140 mM NaCl, 5 mM KCl, 2 mM CaCl2, 1 mM MgCl2, 10 mM D-Glucose, 10 mM HEPES in distilled water). Coverslips were imaged every 1s with 4x4 binning for 340 s on a Zeiss inverted fluorescence microscope with a 10x objective, dichroic LP 409 mirror, BP 340/30 and BP 387/15 excitation and 510/90 emission filters. ZEN Blue software was used for image acquisition and selection of regions of interest (ROIs). ECF was perfused continuously over the cells. After 300s of baseline recording, the cells were perfused with 10μM ATP for 30s. This was followed by a 180s washout and then 1μM capsaicin treatment was added for 30s followed by 180s washout. Neurons were then identified by their responsiveness to 50 mM KCL (30s) and followed by a 30s washout.

Data was analysed using a Matlab script. The amplitude and rate threshold values (at 0.15 and 0.01 for fura2 imaging, respectively) were detected using Excel and a Matlab script. If the maximum amplitude of the calcium trace relative to the baseline (average from 0s to start of first stimulus) is greater than the chosen threshold (0.15units) and the rate of change crosses the chosen threshold (here 0.01) at least once during the analysis period, a response will be

recorded. Only neurons that had a KCL response were considered for analysis. The percentage of cells responding to a certain stimulus was then calculated.

In vitro whole cell patch clamp electrophysiology

For whole cell patch clamp recordings in cultured DRG neurons, data were low-pass filtered at 2 kHz and sampled at 10 kHz. Series resistance was compensated 70%–90% to reduce voltage errors. Patch pipettes (2–4MΩ) were pulled from filamental borosilicate glass capillaries (1.5 mm OD, 0.84 mm ID; World Precision Instruments).

Current clamp

Patch pipettes were filled with internal solution containing (mM): 130 KCl, 1 MgCl2, 5 MgATP, 10 HEPES, and 0.5 EGTA; pH was adjusted to 7.3 with KOH and os- molarity set to 305 mOsm. Extracellular solution contained (mM): 140 NaCl, 4.7 KCl, 1.2 MgCl2, 2.5 CaCl2, 10 HEPES and 10 glucose; pH was adjusted to 7.3 with NaOH and osmolarity was set to 315 mOsm. Resting membrane potential (RMP) was assessed in bridge mode, while firing properties were assessed in current clamp mode. Input resistance (RInput) was calculated from the voltage deflections caused by increasing (Δ20 pA) hyperpolarising current pulses. Capacitance (pF) was measured in voltage-clamp mode. To determine rheobase, cells were depolarised from a holding potential of -60 mV by current steps (50 ms) of increasing magnitude (Δ25 pA) until an action potential was generated. Repetitive firing was assessed by 500 ms depolarising current steps of increasing magnitude (50pA).

Voltage clamp

Patch pipettes were filled with internal solution containing (mM): 120 K+ gluconate, 20 KCl, 2 MgCl2, 10 EGTA, 10 HEPES, 1 CaCl2 and 5 MgATP; pH was adjusted to 7.3 with KOH. In addition, patch pipettes had 2 to 4 MΩ resistance when filled with the internal solution. Extracellular solution contained (mM): 150 Choline-Cl, 5 KCl, 2 CaCl2, 1 MgCl2, 10 HEPES, 0.1 CdCl2 and 10 glucose; pH was adjusted to 7.4 with KOH. The osmolarity of all solutions was maintained at 305 mOsm/L for intracellular solutions and 315 mOsm/L for extracellular solutions. α-Dendrotoxin (α–DTX, Alomone Laboratories) was prepared as a 10,000x stock in H2O and was applied to the bath through the perfusion system. Post-DTX recordings were always made 5 minutes after addition of the drug. All recordings were made at room temperature (19 to 22 °C). Outward currents were elicited by depolarising the membrane potential from −70 to +40mV for 500ms in 10mV increments, following a 1 s pre-pulse

conditioning step to −40mV. The outward current generated at the end of the depolarising pulse was taken as IKD. DTX sensitive currents were obtained by subtracting IKD pre-application of 100 nM DTX from post-drug treatment. Data were analysed by Clampfit 10 software (Molecular Devices) and GraphPad Prism 10.

Extracellular dorsal horn neuron electrophysiology

Mice were initially anaesthetised with 3.5% v/v isoflurane delivered in 3:2 ratio of nitrous oxide and oxygen. Once areflexic, mice were secured in a stereotaxic frame and subsequently maintained on 1.25% v/v isoflurane for the remainder of the experiment (approximately 2-3 hours in duration). Core body temperature was maintained with the use of a homeothermic blanket and respiratory rate was visually monitored throughout. A laminectomy was performed to expose the L3-L5 segments of the spinal cord; mineral oil was then applied to prevent dehydration. Following the identification of WDR neurons the receptive field was stimulated using a wide range of natural stimuli (brush, von Frey filaments – 1, 4, 8 and 15 g and heat – 35, 42, 45 and 48 °C) applied over a period of 10 s per stimulus and the evoked response quantified. The heat stimulus was applied with a constant water jet onto the centre of the receptive field. Ethyl chloride (25 μl) was applied to the receptive field as a noxious evaporative cooling stimulus. Natural stimuli were applied starting with the lowest intensity stimulus with approximately 40 s between stimuli in the following order: brush, von Frey, cold, heat. Electrical stimulation of WDR neurones was delivered transcutaneously via needles inserted into the receptive field after determining responses to natural stimuli. A train of 16 electrical stimuli (2 ms pulses, 0.5 Hz) were applied at three times the threshold current for C-fibre activation. Responses evoked by A- (0–50 ms) and C-fibres (50–250 ms) were separated and quantified on the basis of latency. Neuronal responses occurring after the C-fibre latency band were classed as post-discharge (PD). The non-potentiated response (NPR) and the wind-up (WU) were calculated as: NPR = (action potentials evoked by first pulse) × total number of pulses (16), WU = (total action potentials after 16 train stimulus) − NPR. Rate constant (k) was calculated using non-linear regression analysis. The signal was amplified (x6000), bandpass filtered (low/high frequency cut-off 150/2000 Hz) and digitised at rate of 20 kHz. Data were captured and analysed by a Cambridge Electronic Design 1401 interface coupled to a computer with Spike2 software v4 (CED, Cambridge, UK) with post-stimulus time histogram and rate functions. One to three neurons were characterised per mouse; in total, 16 neurons were characterised from 13 $LGI1^{\mathrm{fl}/Hoxb8-}$ and 16 from 13 $LGI1^{\mathrm{fl}/Hoxb8+}$ mice. Both male and female mice were used aged 8-12 weeks

RNA isolation and cDNA synthesis

Mice were culled using a CO2 chamber. Dissected spinal cord and DRGs were immediately frozen on liquid nitrogen and stored at −80. RNA was isolated using a combination of TriPure (Roche) and a High Pure RNA tissue kit (Roche). Briefly, tissue was homogenized in Tripure using a handheld homogenizer (Cole-Parmer) treated with chloroform and then subjected to column purification before being eluted in RNase free water. Synthesis of cDNA was carried out using Transcriptor reverse transcriptase (Roche), random hexamers (Invitrogen) and dNTPs (Roche).

Quantitative (q)PCR

For analysis of mRNA expression, cDNA (5ng) and primers (0.5µM) were mixed with LightCycler 480 SYBR Green Master (Roche) in a 1:1 ratio and added to white 384 well plates (Roche). Plates were run on a 45-cycle protocol using the LC 480 II system (Roche). Primers were designed using Primer-BLAST (https://www.ncbi.nlm.nih.gov/tools/primer-blast/; Table 2). Primer efficiency and specificity were validated before experimental use. Gene expression for each target primer was normalized against 3 reference genes (18 s, GAPDH and HPRT1) using the delta delta CT method.

RNA Sequencing

Human spinal cord snRNA-seq

Aggregated count data (post QC) and associated metadata (included subtype annotations by barcode) were downloaded from GSE190442[6]. Counts from all annotated neuronal barcodes (3153 barcodes) were converted to a Seurat Object (min.cells = 3, min.features = 200) in R. Data were transformed using `SCTransform` and clustered on the first 30 dimensions for visualization. Barcode annotations were directly extracted from the published metadata.

Human DRG spatial-seq

Spatial-seq data from lumbar hDRG was shared as a Seurat Object from[7] and visualized using the Seurat package (R).

Human DRG, bulk RNA-seq

Using previously published bulk RNA-seq of human DRG, gene counts generated by Ray et al., 2023 using STAR (--*quantMode geneCounts*) were extracted for the neuron-enriched

DRGs for differential hypothesis testing. Data were processed through a standard DESeq2 pipeline with apeglm skrinkage, modelling ~batch (read.length) + pain + sex[8,9]. Significance was determined by an absolute log fold change > 1, and an FDR < 0.05. Data were plotted as quantile-normalized transcripts per million (qnTPM), in line with the published data.

Immunohistochemistry

DRG and spinal cord tissue sections were washed once in PBS and PBS Triton-X (0.3%), before being incubated overnight at room temperature with the respective primary antibodies (Supplementary Table3) diluted in PBS Triton-X (0.3%). Primary antibody was washed off in PBS triton-X (0.3%) and sections incubated with secondary antibodies (Supplementary Table3) at RT for 2-4 hours (Alexa Fluor, Thermo Fisher Scientific). Sections were then washed and cover-slipped. Immunostaining was visualized using a confocal microscope (Axio LSM 700, Zeiss) and images acquired using the Zen black software.

Immunohistochemistry of synaptic proteins

Staining of synaptic proteins was conducted in a similar fashion to that described previously.[10] 30μm sections of mouse lumbar spinal cord were mounted onto gelatin-coated SuperFrost slides, air-dried for 24 hours, rehydrated in PBS containing 0.3% Triton X-100 & 0.1% azide (PBSTxAz) for 5 min then placed in a solution of 50% ethanol, diluted in PBSTxAz containing normal donkey serum (1mg/ml-blocking solution) for 30 min at room temperature. Following three blocking solution washes (10 min each), a heat-mediated antigen retrieval step was conducted by placing sections in Tris-EDTA buffer (10 mM Tris base, 1 mM EDTA, 0.05% Tween-20 at pH = 9.0) for 5 hours at 60 °C in a pre-warmed oven. Slides were then washed with blocking solution (three times, 10 min each) and treated with proteinase K treatment (4 μg/mL) for 10 min at room temperature. Three blocking solution washes (10 min each) were then performed and followed by 10 minutes of pepsin treatment (1 mg/mL, Promega) at 37°C in 0.2M HCl solution with continuous agitation. Following three blocking solution washes (10 min each), sections were incubated with primary antibodies diluted in blocking solution for three days at room temperature. Slides were then washed and incubated for 2-4 hours with the appropriate secondary antibodies. Sections were washed, mounted with vectashield and stored at −20°C prior to confocal imaging. Images were captured on the Zeiss LSM 700, using 405nm, 488nm and 546nm diode lasers. One z stack per section was taken at an interval of 0.29μm across the central portion of the superficial dorsal horn, covering lamina 1-3 dorsoventrally. Approximately 20 optical sections per image were taken for analysis.

In situ hybridization

ISH was carried out using the RNAScope 2.5 RED chromogenic assay kit and by following the manufacturer's instructions (Advanced Cell Diagnostics). Briefly, tissue sections were removed from the −80, allowed to equilibrate to RT and re-hydrated in PBS. Pre-treatment required a hydrogen peroxide step at RT; followed by a protease treatment in a hybridization oven at 40°C. Slides were then incubated with a target probe to detect either mouse *Lgi1*, Adam11, 22 or 23 mRNA or a negative control probe targeting the bacterial gene Dapb at 40°C for 2 hours. Following probe incubation, slides were subjected to 6 rounds of amplification and the probe signal developed via a reaction with fast red. To combine with IHC, tissue sections were then washed with PBS-Tx (0.3%) and subjected to the standard IHC protocol.

Image Analysis

Analysis of ISH signal intensity was calculated using ImageJ software. We segmented the cells using Cellpose 2.0, a generalist algorithm for the segmentation of cellular images.[11] Within Cellpose 2.0, we used a human-in-the-loop pipeline to train a custom model by annotating positive cells on the Cellpose graphical user interface. Cells were determined to be positive for target probe mRNA if signal intensity was 3SDs above negative control readings. Signal intensity was then averaged for all positive cells. For analysis of neuronal activity following formalin (5%) injection, $LGI1^{fl/Hoxb8}$ and control mice were perfused two hours post injection and tissue was incubated with a primary antibody against c-Fos. Neurotrace was used to mark neuronal cell bodies. Superficial lamina (I+II) were defined by IB4 staining to mark lamina IIi. A reference template was used from the Atlas of the Mouse Spinal Cord[12] in combination with IB4 staining to mark deeper lamina (III+IV). C-Fos positivity was determined by eye and the average number of c-Fos positive neurons in the dorsal horn was calculated from 3-5 sections per animal for both ipsilateral and contralateral sides. All quantification was performed with the experimenter blind to genotype.

Synapse analysis

All image analysis was conducted using ImageJ by a blinded investigator. Z stack images were processed in 2D and thresholds of GluA1, GluA4, Synaptophysin and PSD95 were determined using negative controls. For each marker, 11-12 random slices corresponding to 5 different animals per groups were manually thresholded, and the average taken and applied to all images within the same experiment. BIOP-JACoP [13] plugin was used to measure colocalization so that

the amount of AMPA receptors associated with either the PSD95 (post-synaptic marker) or Synaptophysin (pre-synaptic marker) could be determined. Percentages represent mean Mander's coefficients of each animal.

Statistical tests

A Student's t test was used to compare the mean of two groups and when data was not normally distributed a non-parametric test was applied (Mann-Whitney). For behavioural studies over time, patch clamp repetitive firing, voltage-current relationships for $I_{KD,}$ and recordings of dorsal horn neurons to increasing mechanical or heat stimuli a repeated-measures two-way ANOVA was used with posthoc Tukey analysis. For c-Fos and CPP analysis a two-way ANOVA was used with posthoc Tukey analysis. For patch clamp studies cells were used as the experimental unit.

References:

1. Chaplan SR, Bach FW, Pogrel JW, Chung JM, Yaksh TL. Quantitative assessment of tactile allodynia in the rat paw. *J Neurosci Methods*. 1994;53(1). doi:10.1016/0165-0270(94)90144-9
2. Cheng L, Duan B, Huang T, et al. Identification of spinal circuits involved in touch-evoked dynamic mechanical pain. *Nat Neurosci*. 2017;20(6):804-814. doi:10.1038/nn.4549
3. Arcourt A, Gorham L, Dhandapani R, et al. Touch Receptor-Derived Sensory Information Alleviates Acute Pain Signaling and Fine-Tunes Nociceptive Reflex Coordination. *Neuron*. 2017;93(1). doi:10.1016/j.neuron.2016.11.027
4. Hargreaves K, Dubner R, Brown F, Flores C, Joris J. A new and sensitive method for measuring thermal nociception in cutaneous hyperalgesia. *Pain*. 1988;32(1). doi:10.1016/0304-3959(88)90026-7
5. Griggs RB, Bardo MT, Taylor BK. Gabapentin alleviates affective pain after traumatic nerve injury. *Neuroreport*. 2015;26(9):522-527. doi:10.1097/WNR.0000000000000382
6. Yadav A, Matson KJE, Li L, et al. A cellular taxonomy of the adult human spinal cord. *Neuron*. 2023;111(3):328-344.e7. doi:10.1016/J.NEURON.2023.01.007
7. Tavares-Ferreira D, Shiers S, Ray PR, et al. Spatial transcriptomics of dorsal root ganglia identifies molecular signatures of human nociceptors. *Sci Transl Med*. 2022;14(632). doi:10.1126/SCITRANSLMED.ABJ8186
8. Zhu A, Ibrahim JG, Love MI. Heavy-tailed prior distributions for sequence count data: removing the noise and preserving large differences. *Bioinformatics*. 2019;35(12):2084-2092. doi:10.1093/BIOINFORMATICS/BTY895

9. Ray PR, Shiers S, Caruso JP, et al. RNA profiling of human dorsal root ganglia reveals sex differences in mechanisms promoting neuropathic pain. *Brain*. 2023;146(2):749-766. doi:10.1093/BRAIN/AWAC266

10. Dawes JM, Weir GA, Middleton SJ, et al. Immune or Genetic-Mediated Disruption of CASPR2 Causes Pain Hypersensitivity Due to Enhanced Primary Afferent Excitability. *Neuron*. 2018;97(4):806-822.e10. doi:10.1016/j.neuron.2018.01.033

11. Pachitariu M, Stringer C. Cellpose 2.0: how to train your own model. *Nature Methods 2022 19:12*. 2022;19(12):1634-1641. doi:10.1038/s41592-022-01663-4

12. Watson C, Paxinos G, Kayalioglu G, Heise C. Atlas of the Mouse Spinal Cord. *The Spinal Cord*. Published online January 1, 2009:308-379. doi:10.1016/B978-0-12-374247-6.50020-1

13. Bolte S, Cordelières FP. A guided tour into subcellular colocalization analysis in light microscopy. *J Microsc*. 2006;224(3):213-232. doi:10.1111/J.1365-2818.2006.01706.X
